# Network regulation meets substrate modification chemistry

**DOI:** 10.1098/rsif.2022.0510

**Published:** 2023-02-01

**Authors:** Vaidhiswaran Ramesh, Thapanar Suwanmajo, J. Krishnan

**Affiliations:** ^1^ Department of Chemical Engineering, Sargent Centre for Process Systems Engineering, Imperial College London, London SW7 2AZ, UK; ^2^ Institute for Systems and Synthetic Biology, Imperial College London, South Kensington Campus, London SW7 2AZ, UK; ^3^ Department of Chemistry, Faculty of Science, Chiang Mai University, Chiang Mai 50200, Thailand; ^4^ Center of Excellence in Materials Science and Technology, Chiang Mai University, Chiang Mai 50200, Thailand

**Keywords:** network regulation, multisite modification, substrate modification‌, signalling network, negative and positive feedback, protein sequestration, multi-level networks

## Abstract

Biochemical networks are at the heart of cellular information processing. These networks contain distinct facets: (i) processing of information from the environment via cascades/pathways along with network regulation and (ii) modification of substrates in different ways, to confer protein functionality, stability and processing. While many studies focus on these factors individually, how they interact and the consequences for cellular systems behaviour are poorly understood. We develop a systems framework for this purpose by examining the interplay of network regulation (canonical feedback and feed-forward circuits) and multisite modification, as an exemplar of substrate modification. Using computational, analytical and semi-analytical approaches, we reveal distinct and unexpected ways in which the substrate modification and network levels combine and the emergent behaviour arising therefrom. This has important consequences for dissecting the behaviour of specific signalling networks, tracing the origins of systems behaviour, inference of networks from data, robustness/evolvability and multi-level engineering of biomolecular networks. Overall, we repeatedly demonstrate how focusing on only one level (say network regulation) can lead to profoundly misleading conclusions about all these aspects, and reveal a number of important consequences for experimental/theoretical/data-driven interrogations of cellular signalling systems.

## 1. Introduction

Networks represent a pervasive theme across the natural sciences, engineering and the social sciences. They have become a basic way of representing and analysing complex interacting systems/agents/entities in these various disciplines. One of the disciplines where the reality of networks is inescapable is the life sciences. The fundamental unit of life, the cell, relies on a massive, highly sophisticated information processing operation for maintenance and progression of life, and a flexible response to both the external and internal environment. Along with genetic networks, biochemical networks (both signalling and metabolic) represent the heart of information processing in cells.

On the face of it, biochemical networks are networks where the nodes and interactions represent biochemical reactions, and cellular biochemical networks (seemingly) represent an unstructured agglomerate, shaped by evolution to perform specific tasks. However, a closer look reveals that this is not the case. There are recurring network structures and motifs—for instance, different types of feedback and feed-forward regulation which are repeatedly encountered [1–5]. These motifs simply encode key regulatory logic structures in these circuits and are often key drivers for commonly observed behaviours such as switching, oscillations, multi-stability, homeostatic and biphasic responses [3,6–8].

The central component of these networks are proteins. Proteins themselves perform multiple functions, and a key way of establishing functionality of different proteins is by post-translational modifications. The post-translational modifications involve a variety of basic covalent modifications (e.g. phosphorylation, ubiquitation) along with more elaborate multisite modification mechanisms, and represent a distinct axis which is a part of signalling networks. In addition, there are also other regulatory factors at the level of substrate modification, including scaffolding and enzyme–substrate feedback [9–12]. Interestingly, it has been found that post-translational modification of substrates themselves may be capable of generating non-trivial qualitative information processing [13–21].

It is striking to note that while both the network level and the substrate modification level are focal points of many investigations (including, increasingly, data-driven and data-rich approaches [22,23]), they are often studied separately. There is very little systematic systems understanding of how these two levels combine and interact, even though they both are part of the same cellular biochemical networks. This is surprising as their interplay is central to a systems understanding of cellular information processing, being relevant to many aspects of cellular functioning: basic information processing characteristics, behaviour of concrete pathways, the impact of evolution on pathways/networks, the reverse engineering of networks and network engineering in synthetic biology.

An example of the complexity of the substrate modification level is provided by multisite substrate modification (wherein a substrate is reversibly modified at multiple sites by different or common enzymes). Multisite modification is a key constituent of many biochemical networks present alongside network regulation. This includes degradation of cell-cycle protein Sic1 [24,25], degradation of the p53 protein (which itself undergoes numerous modifications [26]) mediated by multisite phosphorylation of MDM2 [27,28], the regulation of insulin (via phosphorylation of SER 312 protein) and IP3 (implicated in Huntington’s disease) via IPMK, both involving potential negative feedback [29–31]. The combination of multisite modification and network regulation underpins important cellular processes, including cell-cycle regulation [32], circadian rhythms [33], inflammation [34] and different diseases [35].

There are many instances in such cases where the interplay of multisite modification and network regulation is a direct focal point of interest. An increasing number of studies focus on both the inference and study the role of the multiple independent feedback regulation in p53 [36–38] and Sic1 proteins [24]. Of special interest is how the network regulation is affected by the modification chemistry of these proteins and how their combination provides functionality to the proteins in the respective pathways. Such interplay is also seen to underscore functioning of key cellular processes such as circadian rhythms (transcription–translational feedback loops [39]) and in the MAPK/ERK pathway [11,40]. The complexity of these networks (both the number and nature of modifications, and the different network regulation present) requires a systematic dissection to understand the contribution of each ingredient to the emergent systems behaviour.

In this paper, we develop a systems framework to investigate the interplay between network regulation (and network structure/topology) and substrate modification biochemistry. We use multisite substrate modification as an exemplar case for substrate modification. At the network level, we examine canonical network structures of feed-forward and feedback regulation, which are broadly encountered.

The basic question we aim to address is: how do the network level and the substrate modification level combine? What does substrate modification contribute to network behaviour and what does the ambient network contribute to substrate modification? We deploy a consolidated approach combining computation, analytical work and semi-analytical approaches to address this and reveal the systems landscape of multi-level interaction.

Addressing this question provides important insights into how the two levels combine in various concrete biochemical networks and allows us to trace the actual origins of a given behaviour (whether from the network, the substrate modification layer or a combination). In so doing, we reveal fundamental constraints associated with working at one level, and how distinct emergent behaviour may arise through a non-trivial confluence of levels. This also has implications for the conceptualization and modelling of cellular networks, the inference of networks from data and assessing the role of evolution. Finally, it provides a framework for multi-level engineering of such networks in synthetic biology.

Our analysis provides a number of key insights and testable predictions regarding the impact of feedback and feed-forward regulation on multisite modification. In the context of double-site modification, this reveals key aspects of this interplay, which may be encountered in specific systems, as referenced above. In addition, by focusing on key recurring structures and building blocks at both the network and substrate modification levels and performing a consolidated analysis, we reveal principles and obtain transparent insights into multi-level interaction. By transparently revealing the origins and reasons for a number of aspects of multi-level interaction, via study of simpler systems, we provide a systems knowledge base which is relevant in different contexts, involving the combination of network regulation and substrate modification. This then provides a distinct and fruitful (and in light of this study, a necessary) vantage point and methodology from which to study specific cases, by engaging with further systems-specific details using this analysis as a platform (see Discussion). In fact specific contexts may be best approached by starting with foundational platforms such as these. We point out that focusing on basic building blocks and network modules/motifs has been a very fruitful approach in cell signalling and information processing generally.

All in all, our analysis creates a bridge between studies (including many experimental studies) which focus on the network level alone and studies which focus on substrate modification alone.

## 2. Models and methods

Our assessment of the interplay of network characteristics and substrate modification biochemistry is studied as follows. Our focus on the substrate modification level is on multisite modification. Multisite modification is capable of distinct information processing characteristics intrinsically, and this has been studied in detail [14,16,41–47]. For the substrate modification biochemistry, we focus on a fairly well-characterized substrate modification system: ordered distributive double-site modification of a protein by a common kinase and common phosphatase (see appendix A). This is because this system is directly encountered in multiple signalling contexts and because it is a building block for substrate modification systems. Focusing on multisite modification/demodification by a common kinase/phosphatase allows us to explore the effect of substrate modification complexity without having to consider the effects of multiple enzymes (and potentially multiple regulatory networks) at this stage (discussed later). We point out that our methodology and specific insights (appropriately interpreted and extended) are also relevant to other multisite substrate modification systems (e.g. random double-site modification with separate/common kinases and separate/common phosphatases), though these systems need dedicated studies of their own for a comprehensive understanding.

The model for the double-site modification system is a widely employed kinetic model (see appendix A) describing individual modifications with the relevant enzymes binding to the substrate, forming a complex, and then dissociating, leaving the modified substrate. The models for the individual modification use standard mass-action kinetic descriptions. This model has been widely studied in the literature.

With regards to the network level, we consider canonical network architectures: feed-forward regulation (both coherent and incoherent) and feedback regulation (both positive and negative). The models of these networks again employ descriptions which are widely used in the literature (see appendix A): each network structure contains different nodes, with interconverting active and inactive forms. A kinetic description of the interconversions in each node as well as the network (node to node) interactions determines the model of the network. The behaviour of these networks is also well characterized.

We analyse systematically the interplay between the network and the substrate modification complexity. We focus on key qualitative information processing characteristics (e.g. biphasic dose–responses, multi-stability—given its importance for switching, oscillations, homeostasis) as these are some of the most basic focal points in modelling, and which promise to provide the sharpest non-trivial insights in relation to experiments.

**Figure 1.**
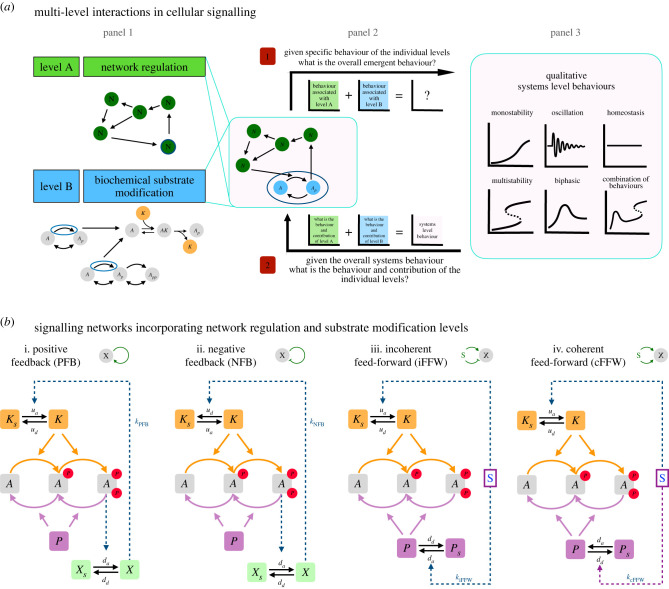
Schematic description of signalling networks involving two levels, the network regulation level and the substrate modification level. (*a*) Panel 1 depicts each of these levels individually; a regulatory network on the one hand and a substrate modification level on the other. Each substrate modification reaction involves enzyme reversibly binding to substrates, creating complexes which results in the modification. Also depicted is a network incorporating both these levels. How these two levels interact is an open question. This can be viewed from two different vantage points (panel 2): 1. Given specific behaviour of the individual levels, what is the overall emergent behaviour? 2. Given the overall systems behaviour, what is the behaviour and contribution of the individual levels? Panel 3 depicts different types of representative behaviours of interest, either for the individual level or the complete system. (*b*) s The different signalling circuits studied in this paper, involving canonical network regulation (feedback and feed-forward) and ordered double-site modification, mediated by a common kinase and common phosphatase, as an exemplar of substrate modification. The panels depict double-site phosphorylation (DSP) systems with (i) positive feedback regulation, (ii) negative feedback regulation, (iii) incoherent feed-forward regulation via a signal and (iv) coherent feed-forward regulation via a signal. The feedback and feed-forward regulation are achieved by promoting activation (or deactivation) of the relevant enzymes.

We consider network regulation overlaid on multisite modification (both realized through generic models; see appendix A) as follows. We regard the entire multisite modification system, along with its associated active and inactive enzymes as a node in the network. Regulation acts by altering the balance between active and inactive enzymes, either via feed-forward regulation (purely by signals) or via feedback regulation (involving a modified substrate); see figure 1 and appendix A. We point out that the resulting model also incorporates a regulation by a signal. The signal regulates either only the balance between active and inactive kinase enzymes (in the cases where we study feedback regulation) or the balance between active and inactive kinases and phosphatases (in the cases where we study feed-forward regulation). Our default choice of signal regulating the kinase simply mirrors the more typical case encountered in signalling networks. Other cases such as signal regulating the phosphatase (in the case of feedback regulation) can also be studied, but that will not be undertaken here. Further details on the computational tools and methodologies are also presented in appendix A.

Ordered double-site modification is known to intrinsically be capable of a range of behaviour such as hyperbolic dose–response characteristics, biphasic responses, bistability and (when enzyme activation is present) oscillatory behaviour [13,14,16,17]. This rich repertoire of behaviour can be traced to the sequestration of enzymes and substrate in multiple complexes. With regard to network behaviour, positive feedback is associated with self-reinforcement, elevation of output, and in the presence of co-operativity, can give rise to bistability [48]. Negative feedback, by contrast, has an attenuative effect, and is implicated in many homeostatic structures, including adaptation as well as (in conjunction with delay) oscillations [49]. Incoherent feed-forward behaviour is a basic way of realizing adaptation while coherent feed-forward networks represent a reinforcement from both regulatory legs.

Understanding the interplay of the network and the substrate modification amounts to addressing the question: what does the substrate modification contribute to network behaviour and what does the network contribute to substrate modification? In each instance, we aim to monitor transitions in qualitative behaviour: for instance, a distinct type of behaviour which is seen in the network as a consequence of substrate modification biochemistry, and transitions in behaviour of multisite modification owing to the network.

We note at the outset that elucidating the interplay of substrate modification and network regulation involves effectively engaging with the associated sets of parameters. This is achieved by a multi-pronged exploration of the underlying models using computational, analytical and semi-analytical approaches, specifically tailored to the questions of interest. Each of these approaches brings complementary insights and new perspectives and taken together, they constitute a consolidated global approach to answer these questions and reveal different facets of the interplay of the levels and their dependence on parameters. This is crucial for revealing the multi-level systems landscape transparently.

Computational results (combining simulations and bifurcation analysis) directly reveal transitions in behaviour and distinct new facets which emerge. In most instances (unless otherwise mentioned), the results obtained are seen widely in parameter space, and computations in multiple parameter regimes reveal this. In some cases, special/unexpected behaviour which is less widespread may be observed and this is noted as such. Analytical work on the other hand is focused on different goals: (i) explaining unexpected new behaviour observed or key transitions in behaviour and (ii) establishing unambiguously the impossibility of certain behaviour/transitions in behaviour. Taken together, this establishes strict boundaries between the presence and absence of certain behaviour. This allows us to make sharp conclusions regarding the origins and key drivers of overall systems behaviour. Such analytical approaches have been applied in different ways: (i) to the full combined system (requiring no assumptions/restrictions on any parameters) and (ii) with some restrictions on the parameter regime of substrate modification (but none on the network). The restrictions could either be on relative enzyme and substrate total amounts or requiring certain enzymatic modifications act in the unsaturated regime. In each case, results demonstrating impossibility of behaviour are obtained independent of all other parameters (see appendix A). Semi-analytical approaches bridge computational and analytical approaches, and in so doing (i) provide a tighter and more transparent elucidation of computational observations and (ii) expand the applicability of analytical approaches by combining with computation in different ways.

## 3. Results

Computational results are presented, followed by a concise summary of analytical/semi-analytical approaches.

### 3.1. Computational results

We consider different possibilities of the behaviour of the multisite modification module in isolation as reference points for evaluating the impact of the network. This includes monotonic dose–responses, bistability, biphasic responses and oscillations. Each of these reference behaviours is represented with a representative parameter set. For each of these reference behaviours, we evaluate the effect of each of the networks, varying key network parameters as needed. Such an approach exploring different combinations of multisite modification behaviour and different network characteristics yields an array of results revealing the landscape of network substrate modification interaction with multiple insights. The results are presented for each of these networks, in turn.

The computational results are underpinned by a broad computational exploration. In this regard, we note the following. (i) The transitions we report are representative, in the sense that the transitions are seen for different parameter sets representing the basal behaviour of the multisite module. (ii) In a few cases, there is a special behaviour which is less widespread and in those cases we note it as such. (iii) We present the noteworthy transitions in behaviour observed computationally. (iv) Many aspects of the computational results, especially the steady-state behaviour, can be explained analytically and rationalized semi-analytically as well and our complementary explorations from these viewpoints provide associated results and approaches.

**Figure 2.**
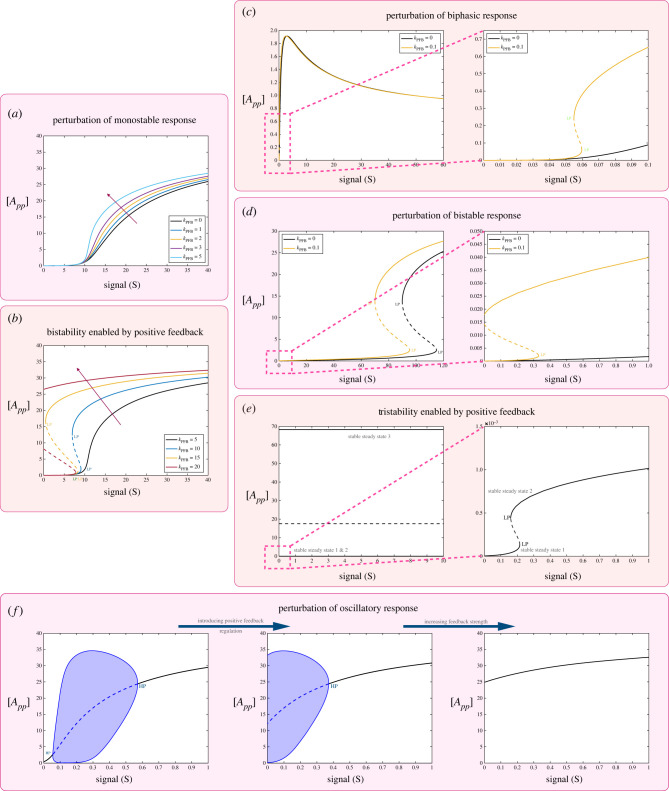
Impact of positive feedback mediated by the fully modified substrate *A*_*pp*_ on the behaviour of the double-site modification system. The results shown demonstrate the effect of positive feedback on different basal characteristics of the double-site modification system. (*a*,*b*) Perturbation of monostable basal response. Panel (*a*) shows increasing positive feedback strength resulting in more switch-like behaviour. Panel (*b*) shows how increasing feedback strength can induce multi-stability in the system (reversible and irreversible). (*c*) Perturbation of biphasic basal response; increasing feedback strength can lead to a combination of multi-stability and biphasic behaviour (region magnified and shown on right-hand plot). (*d*) Perturbation of basal bistable response; increasing feedback strength can lead to distinct regions of multi-stability emerging (region magnified and shown on right-hand plot^1^). (*e*) Feedback can also induce distinct behaviour such as tristability that both the network regulation and the substrate modification levels are otherwise intrinsically incapable of (region magnified and shown on right-hand plot). (*f*) Increasing feedback strength can shift and reduce the range of oscillations leading to its eventual abolishment. (Dashed lines indicate unstable steady states, while solid lines represent stable steady states in the bifurcation diagram. Shaded regions in the bifurcation diagram indicate regions of oscillations, and the blue lines indicate bounds on concentrations during oscillations. HP, Hopf bifurcation; LP, saddle node bifurcation.)

*Positive feedback.* Figure 2 demonstrates different aspects of positive feedback regulation associated with the fully modified phosphoform (see schematic in figure 1*b*).

*Positive feedback can significantly impact switching behaviour and enable the creation of new zones of dose–response characteristics.* Figure 2*a*,*b* demonstrates the impact on monostable threshold behaviour. Increasing the feedback strength can result in a more pronounced (monostable) switch-like behaviour and even result in bistability, which still maintains the threshold-like characteristic. The capacity of positive feedback inducing bistability is also seen in how positive feedback affects multisite modification in a bistable regime (figure 2*d*,*e*). One manifestation of this is the introduction of an additional zone (in the dose–response curve) of bistability, resulting in multiple regimes of bistability. Here, one can associate each distinct bistable zone with the impact of a different factor (intrinsic bistability, feedback). This demonstrates how different zones of dose–response can be engineered and tuned.

*Positive feedback can enable behaviour otherwise inaccessible.* In contrast to generating distinct zones of bistability, positive feedback can also enable tristability (see figure 2*e* and analytical results in electronic supplementary material, file S1). This is noteworthy because it is impossible to obtain tristability both in the multisite modification module in isolation, and in the positive feedback network acting on a single-site modification. Thus, this is an instance of emergent behaviour arising from the combination of multisite modification and positive feedback.

Positive feedback with co-operativity giving bistability is a well-established concept. Co-operativity emerges from basic considerations in such multisite modification, and has been invoked (in concert with positive feedback) to obtain bistability in cell cycle dynamics [50]. The above results indicate other essentially different ways in which multisite modification can reinforce positive feedback.

*Positive feedback as a tool for creating, tuning and combining characteristics.* Positive feedback impacts biphasic behaviour (figure 2*c*) in a specific way: the peak of the biphasic response is maintained, as is the asymptotic response for large signal. The signal value at which the peak is attained is however shifted towards lower signal values and eventually the biphasic response is eliminated. These results are also established analytically (see pp. 90–107, electronic supplementary material, file S1). Positive feedback can also result in biphasic behaviour being combined with bistability. Positive feedback can result in the shifting of the range of oscillations, and a progressive reduction and eventual elimination of the oscillatory range (figure 2*f*).

**Figure 3.**
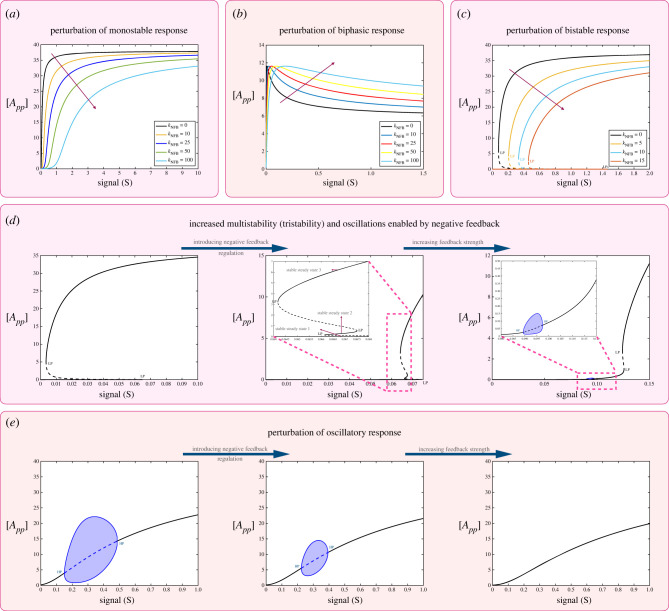
Impact of negative feedback mediated by the fully modified substrate *A*_*pp*_ on the behaviour of the double-site modification system. The results shown demonstrate the effect of negative feedback on different basal characteristics of the double-site modification system. (*a*) Perturbation of monostable basal response; eroding switch-like response. Similar results are observed for a range of monostable basal responses, which are switch-like. (*b*) Perturbation of biphasic response by increasing feedback strength. Results suggest that the peak concentration of *A*_*pp*_ remains the same as feedback strength is changed, while the location of the peak shifts towards higher signal values. (*c*) Negative feedback can impact bistable responses by reducing the range over which they occur. As the feedback strength is increased further, the bistability is destroyed (not shown). (*d*) Other ways in which negative feedback can impact basal bistable behaviour is by the creation of tristability (a behaviour impossible in the basal substrate modification system) as well as the creation of combined oscillatory and multistable responses (magnified regions shown inset). (*e*) Negative feedback can shift and reduce the range of oscillations leading to its eventual abolishment. (Dashed lines indicate unstable steady states, while solid lines represent stable steady states in the bifurcation diagram. Shaded regions in the bifurcation diagram indicate regions of oscillations and the blue lines indicate bounds on concentrations during oscillations. HP, Hopf bifurcation; LP, saddle node bifurcation.)

*Negative feedback.* We examine the case of negative feedback (see schematic in figure 1*b*) through the fully modified phosphoform (figure 3).

*Negative feedback can result in an increase in the maximal number of steady states possible.* Negative feedback can accentuate threshold-like behaviour while diminishing switching behaviour, reducing the amplitude of switching and making the transition more gradual (figure 3*a*). Negative feedback also has the effect of shrinking the range of bistability (figure 3*c*), in the case when the multisite modification system is in the bistable regime. Unexpectedly however, a negative feedback can also result in tristability as seen in figure 3*d*. In fact, it is possible to observe tristability with oscillations: the oscillations may either occur within the zone of multi-stability (as signal is varied), or occur in a signal range where the system is monostable (see electronic supplementary material, file S2, figure S1). The presence of tristability is particularly noteworthy since (i) the multisite modification system in isolation is atmost bistable, and cannot exhibit tristability and (ii) the negative feedback has resulted in an expansion in the (maximal) number of steady states attainable. This feature encountered here is not seen if the modification system has only a single site. The single-site modification system is monostable and negative feedback (with or without an extra covalent modification step) cannot generate bistability (results not shown). Analytical results provide further insights into when a negative feedback creates an expansion of the number of steady states in double-site modification (see p. 13, electronic supplementary material, file S1).

The general expectation of feedback (based on many studies of networks) is that positive feedback can increase the (maximal) number of steady states of a system, while negative feedback plays a dampening/homeostatic role. The ability of negative feedback to increase the maximal number of steady states suggests the need to carefully reassess the role of negative feedback.

Associated experimental observations may be incorrectly inferred as a positive feedback, while in fact being a confluence of negative feedback and modification complexity.

*Feedback regulation can tune biphasic responses while maintaining their amplitude.* The perturbation of the biphasic response (figure 3*b*) shows that the biphasic behaviour appears to be eroded by negative feedback. However, a careful computational examination complemented by analytical work (see pp. 90–107, electronic supplementary material, file S1) reveals that the biphasic behaviour exists as before (and is seen more clearly when larger ranges of signals than in figure 3*b* are considered), and that the amplitude of the peak of the response is fixed, independent of the feedback, as is the asymptotic value for large signal values (implying that the difference between peak and saturation value is also fixed). However, the value of signal at which the peak is attained is progressively pushed to higher signal values. Thus if we focus on a fixed range of signal, negative feedback can eventually eliminate the biphasic response. It is also possible (as feedback strength is increased) for a biphasic peak to be present within a fixed signal range, but the biphasic nature of the curve to be not clearly evident in that range.

Observations from both positive and negative feedback indicate that certain key features (peak and saturation concentration expressed by maximally modified substrate) of biphasic response are robustly maintained, while other features can be varied. This suggests that such feedback could be a valuable tuner of biphasic responses.

*Reassessing the role of negative feedback in generating oscillatory behaviour in networks.* Negative feedback can shrink the range of oscillations, eventually destroying them. This is an instance of how negative feedback which may be seen as helpful in generating oscillations actually works in the exact opposite way. Thus ascribing oscillations observed in data (e.g. figure 3*e* (middle panel)) to the underlying negative feedback would be fundamentally misleading. It reinforces the point that certain emergent effects can result from unintuitive combinations at two distinct levels. This means it is insufficient to study features at only one level (network level say), with the expectation that details at another level (modification level say) could be incorporated after the fact.

*Substrate modification chemistry can enable the creation of mixed feedback regulation.* One of the basic characteristics of a multisite substrate modification system is that there are multiple phosphoforms possible, each capable of being a mediator of feedback. A basic examination of this reveals the distinct feature which emerges. A feedback mediated by the partial phosphoform *A*_*p*_ acts as a mixed positive and negative feedback. This is true whether the nature of the feedback was a mirror of the positive feedback or that of the negative feedback considered above. This simply relies on the fact that the same kinase both produces *A*_*p*_ (from *A*) and converts it (to *A*_*pp*_). *A*_*p*_ activating the kinase results in a mixed feedback, wherein it activates both its production (from A) and conversion to *A*_*pp*_. *A*_*p*_ inactivating the kinase results in a different mixed feedback, wherein it inhibits both its production (from A) and conversion to *A*_*pp*_.

**Figure 4.**
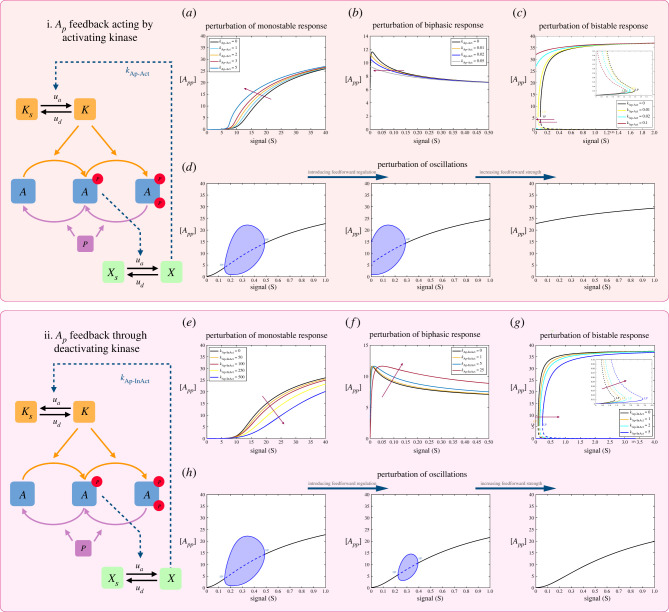
Impact of feedback mediated by the partially modified substrate *A*_*p*_ (through activation or inactivation of kinase) on the behaviour of the double-site modification system. In both these cases, the feedback regulation which ensues is a mixed feedback (combination of positive and negative feedback). (*a*–*d*) Impact of *A*_*p*_ feedback acting by activating kinase on (*a*) monostable response (showing strengthening of switch-like behaviour), (*b*) biphasic response (showing biphasic peak moving to lower signal ranges and the eventual erosion of the behaviour), (*c*) bistable response (introducing irreversible switching)—magnified region inset—and (*d*) oscillatory response (showing abolishment of oscillations with increasing feedback). (*e*–*h*) Impact of *A*_*p*_ feedback acting by deactivating kinase on (*e*) monostable response (showing reduced switch-like behaviour), (*f*) biphasic response (the biphasic peak moving towards higher signal ranges), (*g*) bistable response (increasing the range but reducing the switching amplitude)—magnified region inset—and (*h*) oscillatory response (increasing feedback reduces range of oscillations and leads to its eventual abolishment). The two sets of results above indicate the contrasting effects of mixed feedback regulation by the partially modified substrate and indicate that the specific way in which the mixed feedback is realized has a strong impact on resulting behaviour. (Dashed lines indicate unstable steady states, while solid lines represent stable steady states in the bifurcation diagram. Shaded regions in the bifurcation diagram indicate regions of oscillations and the blue lines indicate bounds on concentrations during oscillations. BP, pitchfork bifurcation; HP, Hopf bifurcation; LP, saddle node bifurcation.)

The feedback from *A*_*p*_ activating the kinase exhibits basic features of a positive feedback (figure 4i); for instance the transition of a basic threshold response to a monostable switch-like behaviour, perturbing bistability to generate irreversible switching, the shifting of the peak of a biphasic response towards low signal levels and the shifting of the oscillatory range towards low signal values and eventually destroying it. We have not however observed the creation of tristability with such a feedback. In a similar way, the feedback from *A*_*p*_ deactivating the kinase (figure 4ii) erodes potential monostable switch-like behaviour, reduces the amplitude of bistable switching, and shrinks and eventually destroys the range of oscillations. In this case, analytical results reveal that tristability cannot be obtained under certain conditions. Analytical work pinpoints further clear-cut differences between feedback mediated by the fully modified phosphoform and the partially modified phosphoform (see pp. 5–89, electronic supplementary material, file S1).

A greater number of modification sites allows ample scope for such mixed feedback to occur in networks. Mixed feedback (resulting from feedback mediated by partial phosphoforms through the enzymes) can however be eliminated by having distinct enzymes for each modification step. Thus an apparently minor detail (intrinsic to a node at the network level) of modification biochemistry can have a strong impact on the network regulation.

*Feed-forward regulation.* We now consider feed-forward regulation wherein a signal regulates the activation/deactivation of two enzymes simultaneously. For any fixed signal value, at steady state, the system exhibits a response corresponding to the behaviour of the system in isolation (perhaps for a different parameter value; see [15]). Thus the different types of feed-forward regulation in many respects (especially steady-state behaviour) amount to the signal regulating the system, traversing specific paths in the space of isolated system behaviours.

*Incoherent feed-forward regulation.* An incoherent feed-forward regulation applied to a simple covalent modification cycle results in different types of adaptive behaviour—exact adaptation, under specific conditions and inexact adaptation which can manifest as over-adaptation and under-adaptation. What impact does it have on multisite modification?

**Figure 5.**
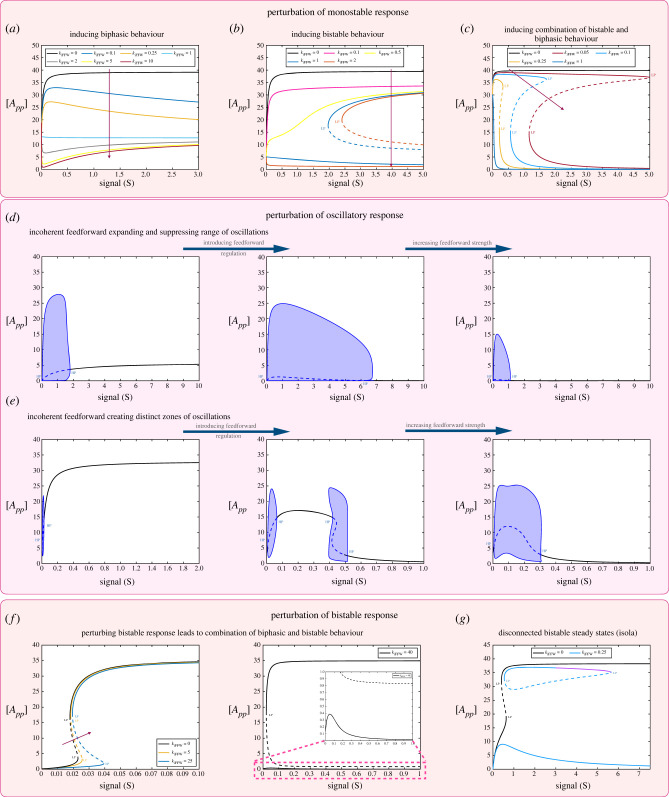
Impact of incoherent feed-forward network regulation on the double-site modification system. In all cases, we vary the kinetic parameter associated with the signal regulation of the phosphatase as a way of probing the impact of incoherent feed-forward regulation and assess its impact on the dose–response curve. Note that if this parameter is 0, there is no incoherent feed-forward regulation. (*a*–*c*) Perturbation of basal monostable behaviour. Panel (*a*) shows how perturbation of a monostable response by feed-forward regulation can generate a sequence of transitions involving generation of biphasic response and its subsequent inversion. Panel (*b*) shows how feed-forward regulation can result in bistable responses. Interestingly, feed-forward network regulation is not associated with multi-stability and this behaviour demonstrates how the substrate modification system can introduce new behaviour to the network. Panel (*c*) shows how feed-forward regulation can lead to a combination of bistable and biphasic response. (*d*,*e*) Perturbation of oscillatory response by feed-forward network regulation. Panel (*d*) shows how feed-forward regulation can both strengthen oscillations (increasing range over which oscillations are observed) and destroy them, while (*e*) shows how feed-forward regulation can lead to distinct regions of oscillations emerging, which can coalesce into a single oscillatory region. (*f*,*g*). Perturbation of basal bistable behaviour. Panel (*f*) shows how basal bistable response can be strengthened by increasing feed-forward strength and can also lead to a combination of biphasic and bistable response (magnified region shown inset). Panel (*g*) shows how basal bistable response can be perturbed to give disconnected closed loop steady-state branches (isola—refer to main text for further discussion). (Dashed lines indicate unstable steady states, while solid lines represent stable steady states in the bifurcation diagram. Shaded regions in the bifurcation diagram indicate regions of oscillations and the blue lines indicate bounds on concentrations during such oscillations. BP, pitchfork bifurcation; HP, Hopf bifurcation; LP, saddle node bifurcation.)

*Incoherent feed-forward regulation can introduce both biphasic and bistable dose–response characteristics.* Incoherent feed-forward regulation (see schematic in figure 1*b*) results in a number of non-trivial transitions (figure 5). If the multisite modification system in isolation exhibits behavioural characteristics of a monostable switch, or even a simple hyperbolic response, then the incoherent feed-forward regulation can result in biphasic behaviour. Furthermore, tuning the relative strengths of the arms of the feed-forward regulation can even reverse a biphasic response with a subsequent increase and plateauing (figure 5*a*). The incoherent feed-forward regulation can also result in the introduction of bistability, which would not have occurred without the signal regulating both kinase and phosphatase. This also shows how a signal regulating an antagonistic enzyme can promote not only biphasic behaviour but bistability as well (figure 5*b*). In fact, it can allow for the creation of a combination of both behaviour together in the dose–response curve (figure 5*c*).

An incoherent feed-forward network is associated with in-built competition, and is, as such, hardly a candidate associated with bistability (which involves reinforcement and co-operative effects). The above results show that (a) this is possible and (b) characterizing the network and the modification system (for the same parameters) in isolation provides no indication of how such behaviour may be generated by incoherent feed-forward regulation.

Incoherent feed-forward regulation can create, merge and separate zones of oscillation. We found that feed-forward regulation could impact oscillations by increasing the range of oscillations, and then dramatically reducing it and even eliminating oscillations (figure 5*d*). Another way in which the feed-forward regulation may impact oscillatory behaviour is by creating distinct regions of oscillations in the dose–response curve which eventually merge (figure 5*e*). This again highlights how feed-forward regulation can enhance robustness of oscillations.

*The creation of isolas.* Incoherent feed-forward regulation can significantly shape and regulate bistable behaviour. This includes an expansion of the range of bistability and the introduction of simultaneous bistable and biphasic behaviour (figure 5*f*). Another distinct outcome is the creation of an isola (an isolated closed curve in the dose–response plot), which is a different manifestation of multi-stationarity, which has not been observed (to our knowledge) thus far in double-site modification (figure 5*g*). Isolas have been observed in different scientific contexts including complicated multisite modification networks [51,52]. They could play a role in biological settings, by creating distinct new states which may not be accessible by conventional switching, but may be realized via strong perturbations of the system.

*Robustness and homeostasis.* Incoherent feed-forward networks are associated with homeostatic responses to signal. If we start with a basal symmetric multisite modification, incoherent feed-forward regulation can be used to generate symmetry-breaking and the creation of absolute concentration robustness (ACR) [53], thus allowing for the network to present a suite of homeostatic responses including both signal homeostasis (itself expanded) and ACR (see electronic supplementary material, file S2; figure S2 and discussion).

#### 3.1.1. Coherent feed-forward regulation

**Figure 6.**
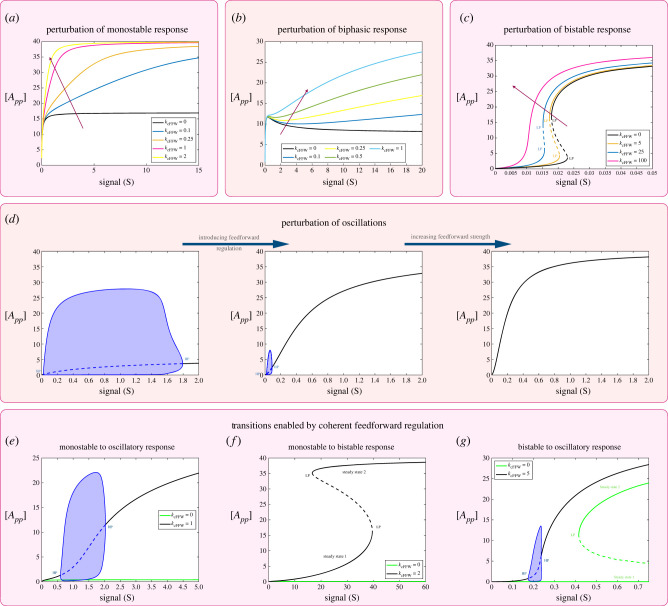
Impact of coherent feed-forward network regulation on the double-site modification system. In all cases, we vary the kinetic parameter associated with the signal regulation of the phosphatase as a way of probing the extent of coherent feed-forward regulation and assessing its impact on the dose–response curve. Note that if this parameter is 0, there is no coherent feed-forward regulation. (*a*) Perturbation of basal monostable response by coherent feed-forward regulation (indicating an elevated response—as may be expected from the reinforcement effects of coherent feed-forward regulation). (*b*) Perturbation of basal biphasic response by coherent feed-forward regulation, demonstrating an erosion of biphasic response and a transition via multiphasic responses to monotonic dose–response. (*c*) Increasing feed-forward regulation can lead to a suppression of bistability. (*d*) Coherent feed-forward regulation can lead to a significant reduction in the range of oscillations and its eventual suppression. (*e*–*g*) Transitions enabled by coherent feed-forward regulation. Panel (*e*) shows how coherent feed-forward regulation can enable a transition from monostable response (green) to an oscillatory response (black). Panel (*f*) shows how monostable response (green) can transition to multi-stability (black), and (*g*) shows how a basal bistable response (green) can be eliminated with the creation of oscillations (black). Panels (*e*–*g*) demonstrate how the simple reinforcement of kinase and phosphatase can result in dramatically different behaviour from the basal dose–response (wherein only the kinase is regulated). This indicates that the effect of coherent feed-forward regulation is not one of simple reinforcement. (Dashed lines indicate unstable steady states, while solid lines represent stable steady states in the bifurcation diagram. Shaded regions in the bifurcation diagram indicate regions of oscillations and the blue lines indicate bounds on concentrations during such oscillations. BP, pitchfork bifurcation; HP, Hopf bifurcation; LP, saddle node bifurcation.)

*Coherent feed-forward regulation is much more than a simple push–pull network.* The simultaneous regulation of kinase and phosphatase (figure 1*b*) to reinforce one other is suggestive, at the outset, of a simple outcome, corresponding to more efficient kinase regulation. However, a closer look reveals that such a picture is simplistic, to say the least. While this simple insight can explain the strengthening of the amplitude of a hyperbolic response (and a monostable switch) as seen in figure 6*a*, we also find the erosion of a biphasic response over the range of input where it occurred (while also presenting multiphasic response in the transition; see figure 6*b*) and the erosion and eventual destruction of bistability (figure 6*c*). As shown in figure 6*d*, the feed-forward regulation can significantly affect the range of oscillations, reducing it and even eliminating it.

*A reinforcement of modification directions is capable of sophisticated control regulation.* The versatility of even the simple coherent regulation is seen in figure 6*e*,*f*, where we see that it can orchestrate multiple types of transitions, from monostable behaviour to both oscillatory and bistable behaviour and also perturb oscillatory behaviour to create bistability; the introduction of bistable behaviour here is in contrast to its tendency to shrink and destroy bistability, seen above. The creation of multiphasic responses from biphasic response is another instance of this (figure 6*b*). This demonstrates the unexpected impact that simple co-regulation of enzymes reinforcing one and other can have.

Taken together, both coherent and incoherent feed-forward regulation afford simple ways of exploring the landscape of the modification chemistry and accessing a range of behaviour, even by varying one (experimentally accessible) parameter.

### 3.2. Analytical results

Analytical results complement the computational results above, providing binary answers to questions underpinning the interplay of the two levels (see electronic supplementary material, file S1, for further details). This section can be skipped by readers not interested in the details.

*Summary of analytical results.* Analytical work focuses on steady-state behaviour and involves systematically reducing the set of model equations at steady state, eliminating most variables and studying the reduced equations. Analytical work either reveals/explains the presence of a behaviour, or proves its absence. In the latter instance, this is established for as broad a range of system parameters as possible: the only restrictions (all in the multisite modification) being either kinetic restrictions in certain steps of enzymatic modification (assumed to be in the unsaturated limit) or total enzyme amounts being much less than total substrate amounts.

A common thread in all the analytical work is the establishing of boundaries between the possibility and impossibility of a given behaviour in either the full system or a simplification/restricted regime thereof. The sharp delineation of structural features which enable or prevent a given behaviour reveals minimal structural ingredients for the manifestation of the behaviour.

*Feedback networks and multi-stability.* We address the fundamental question of how feedback impacts the number of steady states, using two approaches: (a) focusing on enzymatic regimes of specific modification steps being in the unsaturated limit which correspond to the absence of corresponding enzyme–substrate complexes and (b) answering associated questions without making any such simplifying assumptions.

As an example of the first approach, we focus on a series of cases, starting with all enzymatic modification steps in the unsaturated limit (i.e. no complexes) where the behaviour is simple, and then progressively relaxing this assumption. Progressively increasing the number of complexes present (i.e. relaxing the requirement that corresponding modification reactions act in the unsaturated limit) increases the potential complexity (vis-à-vis multi-stability) as seen in the progression of results below. Doing this allows us to isolate key structural requirements for different behaviour (here for simplicity, we assume feedback is directly mediated by the relevant substrate, without an intermediate stage). Complementing the progression below, the various results are also summarized in a table, in both the appendix A (table 1) and the electronic supplementary material (table S2, file S1). (1) If all complexes are removed, the modification system is strictly monostable. Here, a positive feedback mediated by *A*_*pp*_ can generate bistability, but a negative feedback cannot. Furthermore feedback mediated by *A*_*p*_ precludes bistability, whether the feedback activates or inactivates kinase (analogues of positive and negative feedback from *A*_*pp*_)—see pp. 34–51, electronic supplementary material, file S1. (2) Now we consider the case where only one enzyme–substrate complex is present in the substrate modification system. If this involves *A*_*p*_, then bistability is precluded in the double-site modification system (this continues to be true even if both *A*_*p*_ complexes are present; on the other hand, complexes associated with substrates *A* and *A*_*pp*_ readily allow for the possibility of bistability). In this instance, (a) positive feedback from *A*_*pp*_ can result in bistability as expected, but negative feedback is still incapable of doing so; (b) feedback from *A*_*p*_ activating the kinase is capable of inducing bistability if the only complex present is *A*_*p*_*P*, but not if the only complex present is *A*_*p*_*K*. This highlights how the presence of specific complexes in conjunction with feedback can create conditions for bistability, and also highlights the contrast between feedback through *A*_*p*_ and *A*_*pp*_. Feedback mediated by *A*_*p*_ deactivating the kinase will not induce bistability. Both instances of feedback mediated by *A*_*p*_ constitute mixed feedback, but a clear delineation between these two cases is seen in this simplified setting—see pp. 53–77, electronic supplementary material, file S1. (3) In the case where both *A*_*p*_ complexes are present, negative feedback (involving deactivating kinase, mediated by either *A*_*pp*_ or *A*_*p*_) is incapable of inducing bistability (pp. 78–90, electronic supplementary material, file S1). A detailed synthesis of the impact of feedback on these systems where certain complexes are (essentially) absent is discussed in electronic supplementary material, file S1. Taken together, these results reveal the structural requirements in terms of complexes and/or feedback for the creation of bistability.

The second approach is used to examine how feedback can generate tristability, a behaviour not possible in the double-site modification system. In all cases, unless otherwise mentioned, we examine feedback mediated via an extra covalent modification step (see appendix A). (1) We show that a positive feedback mediated by *A*_*pp*_ to the kinase activation can enable tristability, and this can happen even without the extra covalent modification step in the feedback loop (see pp. 7–12 and 19–25, electronic supplementary material, file S1). (2) Analytical work shows how tristability may be generated by negative feedback. In both the positive and negative feedback cases, tristability is possible even when total enzyme amounts are much less than substrate amounts (see pp. 25–29 and 13–18, electronic supplementary material, file S1). (3) We analyse negative feedback mediated by either *A*_*p*_ or *A*_*pp*_, when total enzyme amounts are much less than total substrate amounts, and the extra covalent modification step is absent, and show that tristability is precluded irrespective of system parameters (see p. 29, electronic supplementary material, file S1).

*Regulation via feed-forward networks.* To what extent can feed-forward networks achieve transitions in behaviour between monostability and bistability in multisite modification? (1) At steady state for any fixed signal, the steady state(s) of the system with coherent/incoherent feed-forward regulation can be mapped to the steady state of the isolated double-site modification module, with altered parameters. (2) If the multisite modification system in isolation satisfies an equation (involving catalytic constants) which enables multi-stability (meaning it can be attained for some values of total concentrations of enzymes and substrates), then the introduction of feed-forward regulation (whether coherent or incoherent) will still enable this. (3) There exist criteria for the preclusion of multi-stability for the multisite modification system in isolation. Now using this, we can establish conditions under which a system which was in the ‘exclusion region’ for multi-stability remains so when the signal is varied. This provides an explicit analytic criterion guaranteeing monostability (and prevention of transitions to multi-stability) as the signal is varied. (4) In the same way, we can also establish conditions under which the system can exit the ‘exclusion region’ and provide a basis for determining the minimum signal required to do so (see pp. 108–113 and 118–122, electronic supplementary material, file S1). (5) Using analysis in the low enzyme limit, we demonstrate that the incoherent feed-forward network case can exhibit isolas (explaining computational results), while the coherent feed-forward network will not (and neither will the system in isolation) (see pp. 113–117 and 122–125, electronic supplementary material, file S1).

*Biphasic responses.* Multisite modification systems in isolation can generate biphasic responses (in the maximally modified phosphoform) to varying total kinase amount or signal. We examine how feedback perturbs this. For biphasic responses to change in signal, there is a range of feedback strength over which the biphasic response is maintained (with the peak and saturation value of *A*_*pp*_ remaining fixed). However, a sufficiently strong positive feedback will move the biphasic location to negative signal values, implying an abolishment of the biphasic response. Negative feedback, by contrast, pushes the location of the biphasic response to higher signal values (see pp. 90–107, electronic supplementary material, file S1).

While analytical approaches (especially those which establish results independent of most parameters) provide a number of insights, they are limited by their applicability to certain model simplifications or kinetic regimes. Semi-analytical approaches can expand the applicability of analytical approaches on the one hand, while bringing non-trivial analysis to purely computational explorations.

*Semi-analytical approaches.* Reduction of the steady-state equations results in either single or coupled polynomials, whose coefficients contain all system parameters. While in some cases this may be amenable to analytical work directly, simplifications are often needed. However, with semi-analytical work, it is possible in other instances (at steady state) to combine computation and analytical work to focus on the effect of key network features, which are present as a small number of parameters. The computational component arises here either in the specification of some of the system parameters (e.g. user supplied) and their basal exploration (which is then built upon by analytical work), or in a fuller exploration of the analytical equations obtained, using computation. Either via a study of the polynomial(s) directly or the steady-state Jacobian, it is possible to infer when additional steady states may be created via network regulation, and also when they are guaranteed not to do so. From this, multiple conclusions can be drawn. For instance, by computationally exploring a basal parameter set and finding it to be monostable (a) we can obtain explicit bounds in the network parameter, which ensures that the system remains monostable, (b) we can find ranges of network parameters where additional feasible steady states may be created, and computationally verify when (and if) those may be obtained. (c) Similar conclusions can be drawn if the initial state is multistable. These insights can be obtained for both feedback and feed-forward networks (see pp. 126–133, electronic supplementary material, file S1 for exemplar cases).

## 4. Discussion

**Figure 7.**
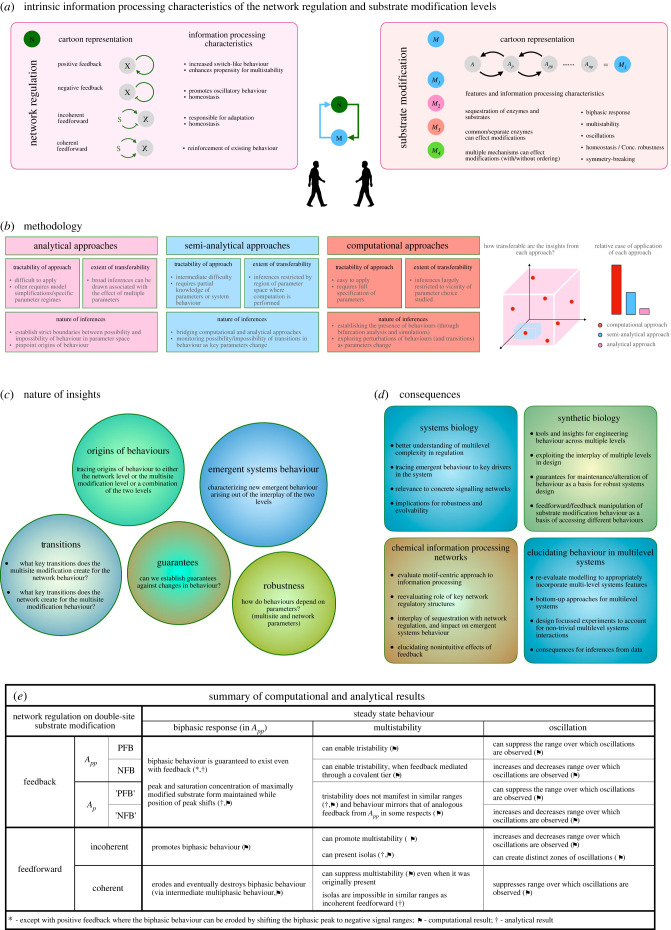
Conceptual summary of the multi-level interaction between network regulation and substrate modification levels studied, the methodology used, and a synthesis of results and insights. (*a*) Network regulation meets multisite substrate modification. The non-intuitive interplay of these two levels is studied here. Network regulation is an integral component of cellular signalling and is responsible for enabling key qualitative behaviours such as switching, homeostasis, multi-stability and oscillations (network topology and nonlinearities are key ingredients here). Multisite substrate modification on the other hand provides proteins specific functionality and is itself capable of complex signal processing capabilities (arising primarily from sequestration effects). (*b*) Methodology. A three-pronged methodology is adopted to tackle the complexity of multi-level interactions. Each approach comes with internal trade-offs between easy applicability and transferability of results across parameter space, and is best suited to provide insights of a specific nature. (*c*) Nature of insights. Our study provides a number of insights ranging from exploring origins of emergent systems behaviour to studying transitions, robustness and guarantees of behaviour exhibited by the overall system. (*d*) Consequences. Our results have consequences in a number of specific contexts. We show non-trivial emergent systems behaviour and non-intuitive roles of network regulation of relevance in systems biology and chemical information processing. By delineating origins of behaviours and studying key drivers of behaviour across multiple levels our study provides results relevant to systems biology. Its relevance to synthetic biology is borne out by the insights it provides to the engineering of multi-level coupled network interactions while guaranteeing robust designs for specific outcomes. The multi-pronged analytical, semi-analytical and computational approach used provides insights to modelling, elucidating and designing of experiments for probing multi-level interactions, and in formulating appropriate data inference methodologies. (*e*) Summary of computational and analytical results.

Multi-levelness is a defining characteristic of living systems, generally, and a hallmark of cell signalling networks. At one level, information is processed through pathways of biochemical reactions, with recurring regulatory structures (e.g. feed-forward/feedback regulation), which (along with associated nonlinearities) contribute to the qualitative complexity. At another level, the functionality of proteins is regulated by protein modifications, and the protein modification status (analogous to a molecular bar-code) determines its function [54]. Qualitative complexity here emerges from ingredients such as sequestration, shared enzymes, scaffolding, multisite modification. The interplay of these levels is thus a theme of broad interest which stems from the fact that proteins, which are key transmitters of information, are often modified in different ways to achieve different functionalities. We investigate this theme in the instance of multisite modification and feed-forward/feedback regulation (see figure 7 for a summary).

Our systems analysis probed the origins and parametric dependence of emergent behaviour/behavioural transitions which resulted from the interplay of the two levels. By employing a combination of computational approaches (directly revealing transitions), analytical work (establishing when transitions and specific behaviour may and may not occur) and semi-analytical work bridging the two approaches, we reveal the multi-level systems landscape with a variety of associated insights. The broader approaches we employ may be fruitfully used for systems dissection of other multi-level systems.

*Transitions between different qualitative behaviour.* Our results show that the ambient network can facilitate transitions to different types of qualitative behaviour in multisite modification. Generally speaking, we find that such transitions are feasible across many parameter sets of the multisite modification module, and different types of behaviour may be obtained depending on the nature (and parameters) of the network. We reveal structural restrictions which may prevent the possibility of transitions from occurring in certain parameter regimes. The network can also induce behaviour not intrinsically observed in substrate modification (e.g. tristability in feedback networks, isolas in incoherent feed-forward networks). Similarly, multisite modification introduces behaviours to the network, which would otherwise not be intrinsically observed (irrespective of parameters), for instance, bistability in feed-forward (both coherent and incoherent) and negative feedback networks. Since this is due to sequestration effects in the multisite module, rather than any explicit positive feedback, we see that bistability emerges in the overall network without any obvious positive feedback present.

*System behaviour and its origins.* Reversing the perspective, and starting from the outcome end, and tracing the ingredients responsible, yields further insights. If a behaviour is intrinsically observed in the multisite modification module, the network can make it either more robust or less robust. If a behaviour is enabled by both multisite modification and the network, the combination is not guaranteed to make the behaviour more robust: in fact, it can lead to the behaviour being abolished (seen in the case of biphasic behaviour and the incoherent feed-forward network, or oscillatory behaviour and negative feedback for instance). This feature (wherein behaviour enabled by each level in isolation cancels each other when levels are combined) implies that simple behaviour of the overall system may not necessarily be associated with simple behaviour of the network and the multisite module (a plausible assumption based on the overall system behaviour). In other cases, the behaviour needs a combination of contributions from both multisite modification and the network, which may however be unintuitive (e.g. tristability arising from negative feedback and multisite modification with intrinsic bistability). These results reveal pitfalls in ascribing experimentally observed behaviour in signalling networks to certain factors without a careful dissection of both substrate modification and network levels.

*Guarantees for maintenance of behaviour.* A complementary aspect of behavioural transitions is establishing guarantees that certain transitions in behaviour do not occur, perhaps for certain ranges of network parameters. Analytical and semi-analytical approaches can provide results which are stronger than and complementary to direct computation. For instance, in exploring potential transitions from single to multiple steady states, it is possible to analytically establish a range of network parameters where a change in the number of steady states is guaranteed not to happen, as an explicit function of the multisite modification parameters. Semi-analytical approaches expand the applicability of such approaches. A similar approach is applied to biphasic dose–responses.

*Chemical modification complexity meets network complexity.* What do the network and substrate modification levels contribute to each other? The network can enable fundamentally new behaviour (e.g. tristability) and establish distinct types of transitions between behaviour intrinsically observed in multisite modification. Feedback regulation can critically depend on the specific phosphoform mediating the feedback. Appropriately designed feed-forward regulation can provide an appealing way of making transitions between different behaviours. This includes accessing a range of behaviour with just one or two experimentally manipulatable signals, something beneficial in synthetic biology. Multisite modification can confer distinct new behaviour to the network including bistability and oscillations in feed-forward networks (otherwise absent). The presence of multisite modification can allow negative feedback regulation to increase the number of possible steady states, something which fundamentally goes against the intuition of negative feedback. More generally the multisite module can cause a particular feedback network to behave like a feedback network of a different type. The root cause for this is sequestration effects in substrate modification, which (a) is internal to a node in the network description and (b) cannot be simply represented as an appropriate sub-network in the network description. The creation of distinctly non-adaptive behaviour in incoherent feed-forward networks is another example of the impact of multisite modification. Overall networks and multisite modification can combine in distinctive and unexpected ways.

*Consequences.* These insights have distinct implications for the ‘modularity’ of such networks and the extent to which they could be understood, studying one level at a time, whether experimentally or theoretically. On the one hand, it indicates the need for focused experiments designed to explicitly probe the interaction of levels (something accessible, since there are plenty of experiments probing each level separately). It also indicates the need to carefully manage and combine data from studies of each of these levels (also see [55]). There are consequences for modelling and theoretical approaches as well. While a motif is a suitable vantage point for investigating core building blocks of networks in general, it will be necessary to investigate motifs which incorporate multi-level information (e.g. core network structures with key building blocks of substrate modification, say). Our studies use models which represent multi-level building blocks, since they explicitly incorporate building blocks at two levels. Thus they represent a bottom-up approach to dissecting the multi-level interaction between substrate modification and network regulation. Such combined theoretical and experimental analysis can provide important insights into when substrate modification may or may not need to be described explicitly in complex networks.

We now discuss the consequences of our results for systems biology, synthetic biology and chemical information processing.

*Systems biology.* Multisite modification is a key constituent of signalling networks, and understanding how such networks function involves understanding the contribution of multisite modification. There are multiple instances of this, for example ERK/MAPK signalling with feedback [56], circadian oscillators [33,39], Sic1 regulation in the cell cycle [24]. There are already multiple studies of MAPK signalling with feedback; multisite modification is believed to be a key driver in the generation of circadian oscillations [39,57]; there are models of specific systems such as Sic1 signalling or Tau protein regulation [24,58]. In such cases, our study provides a systematic platform from which to examine the behaviour of the system, and how it emerges from the contribution of multisite modification. In these cases, there are other aspects of the networks involved as well (e.g. MAPK has an additional layer with double-site modification, circadian oscillators have additional network layers). It is therefore especially important to isolate how different overall behaviour can emerge, and which components/interactions may be key contributors. Our results provide insights and testable predictions into stripped down versions of these systems, and can be used as a template with further augmentation to systematically dissect these systems.

How does our approach apply to other systems, in natural or engineered biology, which may involve the interplay of different kinds of networks and multisite (or other complex) modification systems? Our analysis of ordered double-site modification contributes a number of direct insights into the behaviour in networks, including emergent behaviour, impossibility of behaviour, effects of parameters, essential trends and underlying principles associated with interplay of levels, as well as conceptual and methodological insights. Firstly with regard to other networks (e.g. composite feedback systems, or combinations of feed-forward and feedback regulation), these can be analysed in a similar way building on the results/approach here. With regards to substrate modification, for investigations of ordered double-site modification our results provide insights and a direct platform for expansion and use in other contexts (e.g. p53, MAPK) which allows us to systematically assess when the additional factors present make an essential difference. In other cases with a modest number of modification sites (ordered or random mechanisms), a similar approach can be used; while some of the insights translate to those systems as well, further exploration of the multisite modification, along with the methodology and approach here (including the use of semi-analytical work, complementing computational and analytical work), will be very useful. In all these cases, inputs from experiments for either constraining parameters or testing behaviour are especially useful; in this regard, our approach allows us to develop specific experiments to discriminate between different possibilities (of modification system and network) which give rise to the same systems behaviour. Finally, when the number of sites becomes large, an approach which employs simplifications, informed by the experiments/known biochemistry will be the most useful approach (as it will be for dissecting the modification system in isolation itself), which can then be combined with the approach used here.

A common methodology in systems biology is to infer the underlying networks from data [55,59,60]. Our study (for e.g. results on multi-stability, oscillations and biphasic behaviour) indicates that there are multiple routes to such behaviour which can involve different contributions from multisite modification and the network. In some cases, the behaviour emerges from a complex interplay of the two and may not be adequately captured by a description focusing on the network level alone; in fact, a purely network-level description may result in incorrect inferences drawn about network interactions (e.g. an increase in number of steady states attributed to a positive feedback may in fact be due to a negative feedback, which needs a proper description of the substrate modification layer; oscillations may be attributed to a negative feedback which is actually acting to eliminate oscillations). An appropriate network model needs to account for both the substrate modification chemistry and network interactions, and requires experiments which discriminate between different ways in which the behaviour could be obtained. More generally, our study indicates limitations of using simple networks/motifs and the need for employing the flexibility of multi-level descriptions in the inference process.

Tracing the origins of behaviour to key contributing components is also directly relevant to network robustness and evolvability. Networks in systems biology are products of evolution. How a given network performing an information-processing task emerged is often studied by mimicking the evolutionary process computationally/experimentally starting with simpler structures [61,62]. These studies are done at a fixed level (either network level or chemical modification level), but both are potential sources for evolutionary tinkering. Our analysis suggests a broader question: for specific functions (especially encoded by non-trivial systems behaviour) what are the relative contributions of the network and substrate modification? While the answer could vary from system to system, it might suggest broader strategies used by biology: for example, tinkering with a network as a primary determinant with the substrate modification layer being a modulator. If this is the case, this would be suggestive of a broad design principle. On the other hand, depending on the current stage of evolution, the relative roles may be reversed. Similar issues arise while evaluating robustness of behaviour of these systems: key contributors to robustness could come from either the network, the substrate modification layer or their interplay. Investigating these issues involves first recognizing the possibility of such multi-level contributions and then developing experimental and theoretical methodologies to evaluate different possibilities.

*Synthetic biology.* There are multiple studies which have employed multisite modification in synthetic biology [12,63]. In parallel, manipulation of chemical information processing is being investigated in a variety of cellular and cell-free settings [64,65]. Synthetic biology approaches combining both these elements could provide a richer repertoire for engineering. Our study provides a basis for investigating possibilities of systems behaviour and different ways of exploiting these different aspects in tandem. It could be a starting point for providing a tool-kit for developing designs for chemical modification networks, with substrate modification capability and flexibility which could be especially advantageous. It highlights engineering features of feedback regulation from different phosphoforms—for instance, the natural creation of combined positive and negative feedback, and the tuning of biphasic dose–responses keeping key features fixed. The versatility of feed-forward regulation is demonstrated by showing how (i) it can flexibly access and switch between a multiplicity of different behaviours (using factors which are easy to manipulate experimentally), an appealing candidate for design, and (ii) it can enable a suite of homeostatic responses, including both signal homeostasis and absolute concentration robustness. Finally, our study of different theoretical guarantees to ensure that certain transitions in behaviour do not occur (for certain ranges of network parameters or signals) could be relevant in establishing robustness (especially of qualitative behavioural characteristics) in the design process.

*Chemical information processing.* The networks we examine are examples of complex multi-level information processing networks. Their defining characteristic is that, in a network description, the nodes represent complex mass-conserved systems which are capable of rich behaviour themselves (not easily encapsulable in input–output form). Our study provides multiple instances of the subtlety and complexity of such multi-level systems and challenges in dissecting them. For instance, our analysis reveals new dimensions to basic regulatory structures like negative feedback, and indicates that actual behaviour of regulatory structures may crucially depend on apparently minor components of the regulatory pathway. Future work building on the results presented here will consider multisite modification with different enzymes, which brings in the complexity of different enzymes, different associated networks and cross-talk due to shared substrates. Our studies in this paper (not requiring any essential sequestration effects of the network component in the substrate modification layer, or vice versa) already demonstrate the systems complexity of multi-level interactions. Extending this to examine the effects of such sequestration can build on the current study.

Our study has focused on different aspects of the ‘forward problem’ of how network regulation and multisite modification combine, and the emergent behaviour therefrom. The ‘inverse problem’ of determining the behaviour(s) of the network and multisite modification which enable a particular emergent behaviour is significantly more challenging needing a broad parametric exploration, and to start with, a characterization of different types of behaviour of multisite modification in parameter space. This is an avenue for future work, and can use the present study as a platform.

All in all, the fact that cellular biochemical networks can present complex information processing both at network levels and at the (substrate) modification chemistry level has a range of important and non-trivial consequences. These are critical for understanding different aspects of cellular networks, provide new possibilities for engineering biochemical networks in synthetic biology/chemistry and provide new vistas for investigating complex chemical information processing systems. This paper providing a combination of conceptual, methodological and direct insights provides a platform for exploration in all these contexts.

## Data Availability

The paper results are an analysis of models, performed mathematically and computationally. All the relevant details are provided. (1) All details of models are available in the main text. (2) All computational results are in the main text/electronic supplementary material. (3) Associated mathematical proofs of various statements are performed in Maple and uploaded as a PDF file containing all the details. (4) Other details are presented in the electronic supplementary material. The models which are the basis of the paper are all specified in the Methods section and in the electronic supplementary material, file S1. All parameters used in these models are specified here as well. Additionally all models from the paper are detailed in electronic supplementary material, file S2. A Maple document with relevant analytical proofs is provided as electronic supplementary material (along with a PDF version of this file, electronic supplementary material, file S1, for easy accessibility). Thus all relevant data are contained in this submission. The data are provided in electronic supplementary material [66].

## Footnotes

1 Here and elsewhere in the paper, the thick dashed square/rectangle within a plot represents the broad (not exact) region that is magnified in the adjacent/inset plot.

## Appendix A. Models and methods

Our goal in this paper is to investigate the emergent systems level features arising out of the interplay between the biochemistry of substrate modification and characteristic network regulation.

We focus on the behavioural characteristics and consequences of simple network regulation (positive and negative feedback and coherent and incoherent feed-forward) on commonly encountered substrate modification reactions, multisite substrate modification systems. We discuss the choice of both network regulatory structures and the substrate modification systems below.

*Substrate modification.* As an exemplar of substrate modification, we focus on multisite modification systems. Multisite substrate modification plays a major role in many cellular signalling contexts (and has also been shown to demonstrate different signalling behaviours such as homeostasis, multi-stability and oscillations), and has been investigated extensively both experimentally and theoretically. We employ a representative case of substrate modification: the distributive reversible phosphorylation of substrate on two sites with common enzymes (kinases and phosphatases) effecting phosphorylation/de-phosphorylation on both modification sites with strict ordering (order of phosphorylation is opposite to that of de-phosphorylation). Distributive modification implies that the enzyme disassociates after effecting each modification.

Depending on the choice of chemical mechanism, the presence/absence of ordering of modifications and the nature of enzymes involved, different variations of substrate modification are possible. Multisite phosphorylation (and modification) is possible through other mechanisms. For instance, the modifications could be effected processively (where the enzymes remain attached to the substrate until both modifications have been realized). Mechanisms involving combinations of distributive or processive modifications are also possible. With regard to ordering, there are a spectrum of possibilities ranging from fully ordered mechanisms to completely random mechanisms. Even within ordered mechanisms other types of ordering (leading to cyclic networks) are also possible. Finally, it is also possible to have different enzymes effecting modification/demodification at one or both sites. We focus on the common kinase/common phosphatase, ordered distributive double-site modification case in our study because it is encountered biologically in many instances, is well studied and mathematically well characterized, and exhibits a rich range of information processing behaviour. It also allows us to address the questions of interest without considering the multiplicity of enzymes at this stage.

*Network regulation.* We focus on basic feedback and feed-forward regulatory structures, as they represent the most basic network regulatory structures which are commonly encountered. Feedback regulation is a central feature of cellular networks in general and signalling networks in particular. We explore both positive feedback and negative feedback regulation. As discussed below, when feedback is mediated by a partial phosphoform, this naturally results in a mixed feedback (combined positive and negative feedback) and we examine this as well. We also examine two canonical forms of feed-forward regulation: coherent and incoherent feed-forward networks. The motivation for studying feed-forward networks stems from multiple sources. (i) Feed-forward regulation represents a natural counterpoint to feedback regulation, and a basic form of regulation in its own right. (ii) Feed-forward regulation is the natural extension of simple regulation of one of the enzymes (kinase, say) by the external signal: in fact they include that as a special case. Since enzymes are the key regulators of substrate modification, these feed-forward mechanisms represent the most natural ways of dynamically controlling substrate modification. (iii) Feed-forward regulation by the signal is a simple experimentally accessible way of regulation and controlling system behaviour. It is thus a basic network for consideration in synthetic biology. (iv) Incoherent feed-forward networks underpin homeostasis and adaptation in multiple cellular contexts. (v) Feed-forward networks are recurring motifs in protein networks.

**A.1. Models**

The various networks considered in this study are illustrated in figure 1. These networks involve overlaying of a network regulatory motif on the double-site substrate modification system. The models of the networks involve the models of the two constituent building blocks: (1) regulatory motif and (2) the substrate modification system (both realized through generic models).

*Mathematical model of DSP module.* The models for the reversible double-site modification system are based on widely used descriptions of enzymatic modification of substrates: this involves the enzyme reversibly binding with the substrate to give a complex which then irreversibly converts to give the modified substrate along with enzyme dissociation. An abundance of ATP is implicit in this description. The associated binding, unbinding and the catalytic reactions are described using mass action kinetics. This basic description is applied to every modification/demodification step. Associated with this system of ODEs are also the conservation equations for the substrate and the enzymes. We emphasize that we work with deterministic models throughout and defer spatial and stochastic analysis of these systems to a later study.

This results in the following mathematical description of the double-site modification system as a system of ODEs:

A 1 $$\begin{array}{rl} & \frac{d[A]}{dt}=-k_{b1}[A][K]+k_{ub1}[AK]+k_{4}[A_{\;p}P],\\ & \frac{d[A_{p}]}{dt}=-k_{b2}[A_{p}][K]-k_{b4}[A_{p}][P]+k_{1}[AK]+k_{ub2}[A_{\;p}K]+k_{ub4}[A_{\;p}P]+k_{3}[A_{\;pp}P],\\ & \frac{d[A_{\;pp}]}{dt}=-k_{b3}[A_{\;pp}][P]+k_{2}[A_{\;p}K]+k_{ub3}[A_{\;pp}P],\\ & \frac{d[AK]}{dt}=k_{b1}[A][K]-(k_{ub1}+k_{1})[AK],\\ & \frac{d[A_{\;p}K]}{dt}=k_{b2}[A_{p}][K]-(k_{ub2}+k_{2})[A_{\;p}K],\\ & \frac{d[A_{\;pp}P]}{dt}=k_{b3}[A_{\;pp}][P]-(k_{ub3}+k_{3})[A_{\;pp}P],\\ & \frac{d[A_{\;p}P]}{dt}=k_{b4}[A_{p}][P]-(k_{ub4}+k_{4})[A_{\;p}P],\\ & \frac{d[K]}{dt}=-k_{b1}[A][K]+(k_{ub1}+k_{1})[AK]-k_{b2}[A_{p}][K]+(k_{ub2}+k_{2})[A_{p}K]\\ \;\mathrm{and}\; & \frac{d[P]}{dt}=-k_{b3}[A_{\;pp}][P]+(k_{ub3}+k_{3})[A_{\;pp}P]-k_{b4}[A_{p}][P]+(k_{ub4}+k_{4})[A_{p}P] \end{array}\}$$

with associated conservation equations:

A 2 $$\begin{array}{rl} & A_{\mathrm{Total}}=[A]+[A_{p}]+[A_{\;pp}]+[AK]+[A_{p}K]+[A_{\;pp}P]+[A_{p}P],\\ & K_{\mathrm{Total}}=[K]+[AK]+[A_{p}K]\\ \mathrm{and}\; & P_{\mathrm{Total}}=[P]+[A_{p}P]+[A_{\;pp}P]. \end{array}\}$$

Note that *K* and *P* represent the active form of the kinase and the phoshphatase, respectively. In models where the network regulation (described below) explicitly requires the definition of the active and the inactive form, the activation and deactivation of the enzyme are written as follows in equation (A 3) (where *K*_*s*_ and *P*_*s*_ are the inactive forms of the relevant enzymes) along with suitable augmentations to the conservation equations in equation (A 2). However, when the network regulation does not require an explicit description of activation/inactivation of particular enzymes, all free enzyme is assumed to be active. In equation (A 3), a basal interconversion between active and inactive form of both enzymes is incorporated. In addition the effect of a signal activating the kinase enzyme is also incorporated.

A 3 $$\begin{array}{rl} & \frac{d[K_{s}]}{dt}=-u_{a}S[K_{s}]-u_{\mathrm{beta}}[K_{s}]+u_{d}[K],\\ & \frac{d[P_{s}]}{dt}=-d_{\mathrm{beta}}[P_{s}]+d_{d}[P],\\ & \frac{d[K]}{dt}=-k_{b1}[A][K]+(k_{ub1}+k_{1})[AK]-k_{b2}[A_{p}][K]\\ & \;+(k_{ub2}+k_{2})[A_{p}K]+u_{a}S[K_{s}]-u_{d}[K]+u_{\mathrm{beta}}[K_{s}]\\ \mathrm{and}\; & \frac{d[P]}{dt}=-k_{b3}[A_{\;pp}][P]+(k_{ub3}+k_{3})[A_{\;pp}P]-k_{b4}[A_{p}][P]\\ & \;+(k_{ub4}+k_{4})[A_{p}P]+d_{\mathrm{beta}}[P_{s}]-d_{d}[P]. \end{array}\}$$

*Mathematical model of the regulatory network.* The network regulation motifs are overlaid on the above model of the substrate modification as described below.

For coherent/incoherent feed-forward motifs, a common signal regulating the activation/deactivation of both enzymes simultaneously is incorporated. For coherent feed-forward motifs, this signal activates the kinase while simultaneously deactivating the phosphatase, while in the incoherent feed-forward case, the signal activates both enzymes. In each case, the strength of feed-forward regulation is encapsulated via a kinetic parameter as shown below (*k*_*iFFW*_ or *k*_*cFFW*_). Though other variations of models of feed-forward regulation are possible, the essential elements of feed-forward regulation are already contained in these descriptions and this is sufficient for our study.

*Incoherent feed-forward regulation motif*

A 4
$$\begin{array}{r} \frac{d[K_{s}]}{dt}=-u_{a}S[K_{s}]-u_{\mathrm{beta}}[K_{s}]+u_{d}[K]\\ \;\mathrm{and}\;\frac{d[P_{s}]}{dt}=-k_{iFFW}S[P_{s}]-d_{\mathrm{beta}}[P_{s}]+d_{d}[P]. \end{array}\}$$

*Coherent feed-forward regulation motif*

A 5
$$\begin{array}{r} \frac{d[K_{s}]}{dt}=-u_{a}S[K_{s}]-u_{\mathrm{beta}}[K_{s}]+u_{d}[K]\\ \;\mathrm{and}\;\frac{d[P_{s}]}{dt}=k_{cFFW}S[P]-d_{\mathrm{beta}}[P_{s}]+d_{d}[P]. \end{array}\}$$

In the case of feedback regulation, we have a substrate (either the fully modified form *A*_*pp*_ or the partially modified form *A*_*p*_) contributing to either the activation or inactivation of the kinase. If feedback is mediated by *A*_*pp*_, the former corresponds to positive feedback and the latter to negative feedback. In the case that the feedback is mediated by the partially modified form (*A*_*p*_—either by activating or deactivating the kinase), each case constitutes a mixed feedback. In our models, while incorporating a feedback, we have an intermediate covalent modification tier ($X⇌X_{s}$) which is part of the feedback motif. For simplicity, we assume that the relevant feedback mediator is the sole activator of this intermediate covalent modification tier, though none of the essential results depends on this. We also consider variations of this where the feedback is directly effected by the substrate form through activation or inactivation of the kinase. We note that in any feedback interaction, the substrate (either *A*_*pp*_ or *A*_*p*_) involved in mediating reactions/conversions is not sequestered (this corresponds to the unsaturated limit of enzyme action). In this way, we can examine the primary interplay of the substrate modification and the ambient regulatory network motif without dealing with sequestration effects at intermediate stages.

Thus the mathematical descriptions of the positive feedback and negative feedback motif are as follows.

*Positive feedback regulation motif*

A 6
$$\begin{array}{rl} & \frac{d[K_{s}]}{dt}=-k_{PFB}[X][K_{s}]-u_{a}S[K_{s}]+u_{d}[K]-u_{\mathrm{beta}}[K_{s}],\\ & \frac{d[X_{s}]}{dt}=-d_{a}[M][X_{s}]+d_{d}[X],\\ & \frac{d[X]}{dt}=d_{a}[M][X_{s}]-d_{d}[X]\\ \mathrm{and}\; & X_{\mathrm{Total}}=[X]+[X_{s}]. \end{array}\}$$

*Negative feedback regulation motif*

A 7
$$\begin{array}{rl} & \frac{d[K_{s}]}{dt}=k_{NFB}[X][K]-u_{a}S[K_{s}]+u_{d}[K]-u_{\mathrm{beta}}[K_{s}],\\ & \frac{d[X_{s}]}{dt}=-d_{a}[M][X_{s}]+d_{d}[X],\\ & \frac{d[X]}{dt}=d_{a}[M][X_{s}]-d_{d}[X]\\ \mathrm{and}\; & X_{\mathrm{Total}}=[X]+[X_{s}]. \end{array}\}$$

In the above, [M] is either *A*_*p*_ or *A*_*pp*_ depending on the specific feedback considered.

Using such a description, the models of the full system can be constructed. Variations of these models considered in the study can also be constructed in a similar manner and the detailed description of these can be found in the electronic supplementary material (Maple file).

As illustrative examples, the full model description of positive feedback from *A*_*pp*_ (Model FB: *A*_*pp*_ PFB) and incoherent feed-forward regulation (Model FF: Inc) is provided below. In the positive feedback model below, all phosphatase is assumed to be active.

A 8 $$\begin{array}{rl} Model\;FB:A_{pp}\;PFB\\ & \frac{d[A]}{dt}=-k_{b1}[A][K]+k_{ub1}[AK]+k_{4}[A_{\;p}P]\\ & \frac{d[A_{p}]}{dt}=-k_{b2}[A_{p}][K]-k_{b4}[A_{p}][P]+k_{1}[AK]+k_{ub2}[A_{\;p}K]+k_{ub4}[A_{\;p}P]+k_{3}[A_{\;pp}P]\\ & \frac{d[A_{\;pp}]}{dt}=-k_{b3}[A_{\;pp}][P]+k_{2}[A_{\;p}K]+k_{ub3}[A_{\;pp}P]\\ & \frac{d[AK]}{dt}=k_{b1}[A][K]-(k_{ub1}+k_{1})[AK]\\ & \frac{d[A_{\;p}K]}{dt}=k_{b2}[A_{p}][K]-(k_{ub2}+k_{2})[A_{\;p}K]\\ & \frac{d[A_{\;pp}P]}{dt}=k_{b3}[A_{\;pp}][P]-(k_{ub3}+k_{3})[A_{\;pp}P]\\ & \frac{d[A_{\;p}P]}{dt}=k_{b4}[A_{p}][P]-(k_{ub4}+k_{4})[A_{\;p}P]\\ & \frac{d[K]}{dt}=-k_{b1}[A][K]+(k_{ub1}+k_{1})[AK]-k_{b2}[A_{p}][K]\\ & \;+(k_{ub2}+k_{2})[A_{p}K]+k_{PFB}[X][K_{s}]+u_{a}S[K_{s}]-u_{d}[K]+u_{\mathrm{beta}}[K_{s}]\\ & \frac{d[P]}{dt}=-k_{b3}[A_{\;pp}][P]+(k_{ub3}+k_{3})[A_{\;pp}P]-k_{b4}[A_{p}][P]+(k_{ub4}+k_{4})[A_{p}P]\\ & \frac{d[K_{s}]}{dt}=-k_{PFB}[K_{s}][X]-u_{a}S[K_{s}]+u_{d}[K]-u_{\mathrm{beta}}[K_{s}]\\ & \frac{d[X_{s}]}{dt}=-d_{a}[A_{\;pp}][X_{s}]+d_{d}[X]\\ \mathrm{and}\; & \frac{d[X]}{dt}=d_{a}[A_{\;pp}][X_{s}]-d_{d}[X] \end{array}\}$$

with associated conservation equations:

A 9 $$\begin{array}{rl} & A_{\mathrm{Total}}=[A]+[A_{p}]+[A_{\;pp}]+[AK]+[A_{p}K]+[A_{\;pp}P]+[A_{p}P]\\ & K_{\mathrm{Total}}=[K_{s}]+[K]+[AK]+[A_{p}K]\\ & P_{\mathrm{Total}}=[P]+[A_{p}P]+[A_{\;pp}P]\\ \mathrm{and}\; & X_{\mathrm{Total}}=[X]+[X_{s}]. \end{array}\}$$

**Model FF: Inc**

A 10 $$\begin{array}{rl} & \frac{d[A]}{dt}=-k_{b1}[A][K]+k_{ub1}[AK]+k_{4}[A_{\;p}P]\\ & \frac{d[A_{p}]}{dt}=-k_{b2}[A_{p}][K]-k_{b4}[A_{p}][P]+k_{1}[AK]\\ & \;+k_{ub2}[A_{\;p}K]+k_{ub4}[A_{\;p}P]+k_{3}[A_{\;pp}P]\\ & \frac{d[A_{\;pp}]}{dt}=-k_{b3}[A_{\;pp}][P]+k_{2}[A_{\;p}K]+k_{ub3}[A_{\;pp}P]\\ & \frac{d[AK]}{dt}=k_{b1}[A][K]-(k_{ub1}+k_{1})[AK]\\ & \frac{d[A_{\;p}K]}{dt}=k_{b2}[A_{p}][K]-(k_{ub2}+k_{2})[A_{\;p}K]\\ & \frac{d[A_{\;pp}P]}{dt}=k_{b3}[A_{\;pp}][P]-(k_{ub3}+k_{3})[A_{\;pp}P]\\ & \frac{d[A_{\;p}P]}{dt}=k_{b4}[A_{p}][P]-(k_{ub4}+k_{4})[A_{\;p}P]\\ & \frac{d[K]}{dt}=-k_{b1}[A][K]+(k_{ub1}+k_{1})[AK]-k_{b2}[A_{p}][K]\\ & \;+(k_{ub2}+k_{2})[A_{p}K]+u_{a}S[K_{s}]+u_{\mathrm{beta}}[K_{s}]-u_{d}[K]\\ & \frac{d[P]}{dt}=-k_{b3}[A_{\;pp}][P]+(k_{ub3}+k_{3})[A_{\;pp}P]-k_{b4}[A_{p}][P]\\ & \;+(k_{ub4}+k_{4})[A_{p}P]+k_{iFFW}S[P_{s}]+d_{\mathrm{beta}}[P_{s}]-d_{d}[P]\\ & \frac{d[K_{s}]}{dt}=-u_{a}S[K_{s}]-u_{\mathrm{beta}}[K_{s}]+u_{d}[K]\\ \mathrm{and}\; & \frac{d[P_{s}]}{dt}=-k_{iFFW}S[P_{s}]-d_{\mathrm{beta}}[P_{s}]+d_{d}[P] \end{array}\}$$

with associated conservation equations:

A 11 $$\begin{array}{rl} & A_{\mathrm{Total}}=[A]+[A_{p}]+[A_{\;pp}]+[AK]+[A_{p}K]+[A_{\;pp}P]+[A_{p}P]\\ & K_{\mathrm{Total}}=[K_{s}]+[K]+[AK]+[A_{p}K]\\ \mathrm{and}\; & P_{\mathrm{Total}}=[P_{s}]+[P]+[A_{p}P]+[A_{\;pp}P]. \end{array}\}$$

**A.2. Methods of analysis**

As mentioned in the main text, we use three broad classes of approaches in our analysis: (a) computational approaches combining numerical simulation and bifurcation analysis, (b) analytical approaches and (c) semi-analytical approaches. These three approaches converge to provide key and robust insights about the interplay of the network regulation and the substrate modification, including binary answers to the presence, absence and enabling of behaviours, and the characterization of parameter space for this purpose. All in all, it provides a basis for revealing the systems landscape of the interaction between the network and multisite substrate modification.

The qualitative systems behaviour is a basic focal point of interest both experimentally and theoretically. Consequently, our focus is on qualitative behaviour, and in particular transitions in qualitative behaviour arising from the interplay of network and substrate modification.

We study this by focusing on qualitative transitions which occur due to the network regulation structure being overlaid on the multisite modification module. In this regard, we note at the outset that broadly three types of qualitative effects can be observed in the behaviour of the multisite substrate modification system with the introduction of the network regulation (both described using nominal parameter values): (a) no change, (b) a transition to some behaviour which can be intrinsically exhibited by the multisite modification module at a different parameter regime and (c) a transition to behaviour which cannot be intrinsically observed by the multisite modification module (even including enzyme activation). In our analysis, we aim to assess the interplay of the network and substrate modification, which involves an assessment of the roles of parameters of both the network and substrate modification.

*Computational analysis.* The computational analysis reveals transitions in behaviour using numerical simulations and bifurcation analysis. To do this, we start with well defined qualitative basal behaviour (of the substrate modification in isolation)—e.g. monostable switch-like behaviour, bistable behaviour, biphasic dose–responses and oscillatory behaviour. The analysis then reveals the transitions in behaviour which emerge as a consequence of the network regulation, and we report non-trivial instances of this. Thus such analysis provides an affirmative answer to the question as to whether certain new behaviour and transitions in behaviour are possible. In analysing the presence of transitions in behaviour, we establish patterns of transition in behaviour which are robust in the sense that they can be recapitulated with multiple different basal parameter sets. We also report new and notable/special behaviour which may be obtained, which is less widespread, but whose presence itself is unexpected, and in those cases, note it as such. While computational results establish new behaviours and transitions, they by themselves cannot establish the *impossibility* of either transitions or new behaviour.

All models are numerically simulated using the Matlab ordinary differential equation solver ode15s [67]. Matlab models are cross-validated (in multiple parameter regimes) by comparing the results with simulations in COPASI [68] which requires only specification of network and parameters. Bifurcation analysis is performed in MATCONT [69,70], which uses the Matlab models.

*Analytical work.* The goal of our analytical work is to obtain binary answers regarding the possibility/impossibility of behaviours, transparently accounting for parameters. At the outset, we note that the multisite modification system is capable of exhibiting a fairly rich set of behaviour, and also that the characterization of the parameter space for such behaviour is at best only partially achieved. Noting this and the above points, we use analytical work to (i) establish unambiguously the possibility (or impossibility) of behaviour (or transitions to behaviour) in the combined system which would be impossible intrinsically in the multisite modification system and (ii) establish the possibility (or impossibility) of transitions in the complete system, for the multisite modification system in either a specified subset of parameter space, or with some simplifications (e.g. elimination of certain complexes, which correspond to certain modifications occurring in the unsaturated limit). Implicit in this approach is the fact that the specified parameter subspace or simplifications allows us to unambiguously characterize the possibility/impossibility of the behaviour of interest. Our focus here is on biphasic dose–responses or multi-stability.

*Parameters and analytical work.* We now discuss the role of parameters in our analytical work. For this purpose, it is worth noting that parameters fall into three categories: kinetic parameters of multisite modification, total substrate and enzyme amounts in multisite modification (easy to experimentally manipulate) and network parameters. Analytical work falls into two broad categories, establishing behaviour or ruling it out.

We first discuss analytical approaches for ruling out the possibility of certain behaviour/transitions, noting that analytical approaches are often the only way to definitively answer such questions. A common thread in our approach is to try to establish results which are independent of as many parameters as possible. In all such cases, we establish results independent of network parameters. Our results include analytical conclusions of different types. (i) Establish absence of behaviour independent of all substrate modification parameters (both kinetic parameters and species amounts). This means that such results are established independent of all system parameters. (ii) Results which are established in a regime of parameters associated with species amounts. Here, we typically use the case of total enzyme amounts being much less than total substrate, but establish results completely independent of all kinetic modification parameters. (iii) Results which are established by placing some restriction on the kinetic modification parameters. This is usually done by focusing on instances where one or more enzymatic modification steps is in the unsaturated regime. In such instances, the relevant enzyme substrate complex(es) is essentially absent, but results are established independent of other kinetic parameters and total species amounts.

Analytical work to establish the presence of certain behaviour typically places some restrictions on either kinetics of modification (as discussed above) or species amounts (typically enzyme amounts much less than substrate amounts). Then upon analytical exploration of the equations we are able to transparently see how certain behaviour may be possible, and identify parameter sets for both substrate modification and the network which establish that. These results complement purely computational demonstrations of the behaviour being observed. In this regard, we also note that through a combination of ruling out behaviour (in certain kinetic regimes, where particular complexes are absent) and demonstrating the presence of the behaviour analytically, we establish sharp boundaries between the absence and presence of certain behaviour, identifying minimal structural features required for the behaviour to manifest.

*Semi-analytical work.* The analytical approach above often relies on certain simplifications of the system or restrictions in the parameter space. This allows us, where possible, to characterize the basal behaviour in an unambiguous manner. We complement this with semi-analytical approaches which can be used for instance as follows. (a) Starting with a basal parameter set, one can computationally characterize the behaviour of the system, and then use analytical approaches to establish conditions under which certain transitions may be enabled or impossible. As an example, if the basal parameter set corresponds to monostability, we can establish ranges of network parameters where this remains the case. (b) Even in places where analytical work established the possibility of transitions, this can be used to obtain information about parameters of the network required to establish a transition. In general, the semi-analytical approach can work in different ways: (a) start with initial computations, and use the emerging results along with analytical work to draw conclusions and (b) start with analytical work, which then needs computations in specific instances to draw conclusions. Illustrative examples of such semi-analytical approaches to provide key insights into system behaviour are shown in the electronic supplementary material (for both feedback and feed-forward networks). Specifically, we show how semi-analytical approaches can be used to understand the role of feed-forward and feedback strength in enabling and disabling multi-stationarity in relevant models.

We make a further comment about semi-analytical work and analytical work. In our analytical work, we aim to establish results which may be independent of most (or possibly all) parameters. In addition to restrictions in being able to carry out such analytical work, we also note that obtaining such broad results may not be possible in many instances, simply because the system behaviour may not conform to such a structure. In such a case, a specification of the basal parameter set can allow for the establishment of specific results (analytically), which depend on the specification of the basal parameter set.

The detailed mathematical proofs for the various assertions (using analytical and semi-analytical approaches) is carried out in the computational software Maple [71]. A PDF version of this file is also provided as electronic supplementary material, file 1, for easy accessibility. The various results generated in this study are detailed in table 1, along with references to the location (page numbers) of analytical/semi-analytical results within the electronic supplementary material, file S1, and references to relevant computational results presented in the figures.

**Table 1.** Summary of analytical results and references to their location in electronic supplementary material, file 1 (Maple PDF).

model	network	brief description of analytical result	page and ref.	figure ref.
feedback-reduced models				
**no complex**				
*DSP (no complex)*	*none*	*absence of multi-stationarity*	p. 34	—
DSP (no complex)	*A*_*pp*_ PFB (direct)	presence of multi-stationarity (bistability)	p. 36	—
	*A*_*pp*_ NFB (direct)	absence of multi-stationarity (bistability)	p. 39	
	*A*_*p*_ PFB (direct)	absence of multi-stationarity	p. 43	
	*A*_*p*_ NFB (direct)	absence of multi-stationarity	p. 47	
	*A*_*pp*_ PFB (cascaded)	presence of multi-stationarity (bistability)	p. 37	
	*A*_*pp*_ NFB (cascaded)	absence of multi-stationarity (bistability)	p. 41	
	*A*_*p*_ PFB (cascaded)	absence of multi-stationarity	p. 45	
	*A*_*p*_ NFB (cascaded)	absence of multi-stationarity	p. 49	
**with *AK* complex**				
*DSP (*AK* complex)*	*none*	*presence of multi-stationarity (bistability)*	p. 53	—
DSP (*AK* complex)	all types	presence of multi-stationarity (bistability)		
**with *A*_*p*_*K* complex**				
*DSP (*A*_*p*_*K* complex)*	*none*	*absence of multi-stationarity*	p. 55	—
DSP (*A*_*p*_*K* complex)	*A*_*pp*_ PFB (direct)	presence of multi-stationarity (bistability)	p. 60	
	*A*_*pp*_ NFB	absence of multi-stationarity (bistability)	p. 69	
	*A*_*p*_ PFB	absence of multi-stationarity	p. 65	
	*A*_*p*_ NFB	absence of multi-stationarity	p. 73	
**with *A*_*pp*_ complex**				
*DSP (*A*_*pp*_P complex)*	*none*	*presence of multi-stationarity*	p. 56	—
DSP (*A*_*pp*_*P* complex)	all types	presence of multi-stationarity (bistability)	p. 55	
**with *A*_*p*_*P* complex**				
*DSP (*A*_*p*_P complex)*	*none*	*absence of multi-stationarity*	p. 58	—
DSP (*A*_*p*_*P* complex)	*A*_*pp*_ PFB (direct)	presence of multi-stationarity (bistability, in the enzyme limiting regime)	p. 62	
	*A*_*pp*_ NFB	absence of multi-stationarity	p. 71	
	*A*_*p*_ PFB	presence of multi-stationarity (bistability)	p. 67	
	*A*_*p*_ NFB	absence of multi-stationarity	p. 75	
**with both *A*_*p*_ complex**				
*DSP (both *A*_*p*_ complex)*	*none*	*presence of multi-stationarity*	p. 78	—
DSP (both *A*_*p*_ complex)	*A*_*pp*_ PFB (direct)	presence of multi-stationarity (bistability)	p. 80	
	*A*_*pp*_ NFB	absence of multi-stationarity	p. 84	
	*A*_*p*_ PFB	presence of multi-stationarity (bistability)	p. 82	
	*A*_*p*_ NFB	absence of multi-stationarity	p. 86	
**feedback: full models with direct feedback**				
*DSP*	*none*	*absence of tristability*	Conradi *et al.* [16]	
DSP (full system)	*A*_*pp*_ PFB (direct)	presence of increased multi-stationarity (tristability)	p. 19	—
	*A*_*pp*_ PFB (direct)	presence of increased multi-stationarity (tristability, in the enzyme limiting regime)	p. 19	
	*A*_*pp*_ NFB (direct)	absence of increased multi-stationarity (enzyme limiting regime)	p. 25	
	*A*_*p*_ NFB (direct)	absence of increased multi-stationarity	p. 29	
**feedback: full models with cascaded feedback**				
DSP (full system)	*A*_*pp*_ PFB (cascaded)	presence of increased multi-stationarity (tristability)	p. 7	figure 2
	*A*_*pp*_ PFB (cascaded)	presence of increased multi-stationarity (tristability in the enzyme limiting regime)	p. 7	
	*A*_*pp*_ NFB (cascaded)	presence of increased multi-stationarity (tristability)	p. 13	figure 3
	*A*_*pp*_ NFB (cascaded)	presence of increased multi-stationarity (tristability in the enzyme limiting regime)	p. 13	
**feedback: full models with direct feedback**				
**effect of feedback on biphasic dose–response with signal**				
*DSP (full system)*	*none*	*guaranteed biphasic response when kinetic constraints (involving catalytic constants) are satisfied*	p. 90	figures 2–4
DSP (full system)	any except *A**_pp_* PFB	if the isolated system presents with biphasic dose–response, the system with FB will too	p. 94	figure 4
	any	features of biphasic response such as peak *A*_*pp*_ concentration, and saturation value are preserved with feedback		
	any	the signal concentration when biphasicbehaviour manifests (peak) shifts		figure 4
	*A*_*pp*_ PFB	peak signal concentration shifts to lower ranges		figure 2
	*A*_*pp*_ NFB	peak signal concentration shifts to higher ranges		figure 3
**feed-forward: full models with kinase and phosphatase activation**				
DSP	incoherent FF	mapped behavioural landscape of the system to that of the isolated system with no FF	p. 108	figure 5
		derive modified kinetic constraint guaranteeing and promoting multi-stability		
		presence of isolas (full system)		
		presence of isolas (enzyme limiting regime)		
	coherent FF	mapped behavioural landscape of the system to that of the isolated system with no FF	p. 118	figure 6
		derive modified kinetic constraint guaranteeing and promoting multi-stability		
		absence of isolas (full system)		
**semi-analytical approaches**				
DSP	incoherent FF	role of feed-forward strength in enabling and disabling multi-stationarity	p. 127	—
DSP	*A*_*pp*_ PFB	role of feedback strength in increasing multi-stationarity	p. 130	—

*Parameters.* The parameters for the models illustrated above fall into two categories, those of the multisite modification (kinetic parameters and total amounts of the enzymes and substrate) and those related to the network. In general our computational results fall into two categories: (i) the demonstrations of transitions in behaviour and (ii) the emergence of new kinds of behaviour. This is investigated by starting with a basal parameter set for the multisite modification (which results in certain basal behaviour), and then varying key parameters in the network. We approach the parameters for the multisite modification as follows. The kinetic parameters of modification play a crucial role in determining the behaviour of multisite modification, and this has been characterized in many instances in the literature [16]. We set all enzyme–substrate unbinding constants equal as this does not in any way restrict the range of behaviour which can be obtained. The remaining parameters are catalytic constants and enzyme–substrate binding constants for which we choose representative values within reasonable ranges based on existing studies in the literature. Note that since we use mass action kinetic description for the modification, we make no assumption regarding regimes of enzyme action and the results are thus broadly representative of features of the substrate modification system. We also ensure that the parameter choices are not close to any bifurcation point, and thus we can expect a reasonable neighbourhood of the basal parameter set where the basal behaviour is observed. We also choose enzyme and substrate amounts comparable to one another. In this manner, we choose basal parameter sets for the substrate modification. The parameters related to the network are varied (both in our bifurcation analysis and simulations) to study the behaviour of the network with varying strengths of feedback/feed-forward regulation. In computational work, when a certain computational result is obtained, we check that a similar result can be obtained by a different set of parameters of the multisite modification, to ensure that the behaviour is not in any way restricted to the neighbourhood of the original parameter choice. Parameters in the context of analytical work are discussed in the methodology for analytical work.

We emphasize that our consolidated approach merging computational, analytical and semi-analytical approaches provides a number of crucial insights into how system parameters affect system behaviour.

The focus in this paper was on investigating the interplay of substrate modification complexity (exemplified by ordered double-site substrate modification systems) and network regulation (exemplified by canonical feed-forward and feedback networks). A dedicated systems methodology combining computational, analytical and semi-analytical approaches is used to unravel the interplay of network regulation (complexity arising from network structure and nonlinearity) and substrate modification (complexity arising from species sequestration). As such this approach is directly applicable in many related multi-level systems. For example, the interplay of other networks and ordered double-site modification, the interplay of networks and substrate modification realized by random modification systems (with either common/separate kinases and common/separate phosphatases) and/or a greater number of modification sites. Finally, such a multi-pronged approach is especially suitable for conceptualizing, dissecting and engineering other systems where multi-level interactions are a key determinant of emergent behaviour.

**A.3. Parameter values**

*Figure 2: Role of positive feedback from the fully modified substrate (*A*_*pp*_) in influencing intrinsic double-site modification network behaviour.*

A. *u*_*a*_ = 0.1, *u*_*d*_ = 100, *u*_beta_ = 0.01, *d*_*a*_ = 0.01, *d*_*d*_ = 1, *k*_1_ = 1, *k*_2_ = 1, *k*_3_ = 1, *k*_4_ = 1, *k*_*b*1_ = 1, *k*_*b*2_ = 1, *k*_*b*3_ = 1, *k*_*b*4_ = 1, *k*_*ub*1_ = 1, *k*_*ub*2_ = 1, *k*_*ub*3_ = 1, *k*_*ub*4_ = 1, *A*_Total_ = 40, *P*_Total_ = 1, *K*_Total_ = 5, *X*_Total_ = 1, *k*_*PFB*_ = 0, 1, 2, 3, 5.
B. *u*_*a*_ = 0.1, *u*_*d*_ = 100, *u*_beta_ = 0.01, *d*_*a*_ = 0.01, *d*_*d*_ = 1, *k*_1_ = 1, *k*_2_ = 1, *k*_3_ = 1, *k*_4_ = 1, *k*_*b*1_ = 1, *k*_*b*2_ = 1, *k*_*b*3_ = 1, *k*_*b*4_ = 1, *k*_*ub*1_ = 1, *k*_*ub*2_ = 1, *k*_*ub*3_ = 1, *k*_*ub*4_ = 1, *A*_Total_ = 40, *P*_Total_ = 1, *K*_Total_ = 5, *X*_Total_ = 1, *k*_*PFB*_ = 5, 10, 15, 20.
C. *u*_*a*_ = 1, *u*_*d*_ = 100, *u*_beta_ = 0.01, *d*_*a*_ = 1, *d*_*d*_ = 1, *k*_1_ = 1, *k*_2_ = 0.01, *k*_3_ = 10, *k*_4_ = 1, *k*_*b*1_ = 1, *k*_*b*2_ = 1, *k*_*b*3_ = 1, *k*_*b*4_ = 1, *k*_*ub*1_ = 1, *k*_*ub*2_ = 1, *k*_*ub*3_ = 1, *k*_*ub*4_ = 1, *A*_Total_ = 40, *P*_Total_ = 1, *K*_Total_ = 70, *X*_Total_ = 5, *k*_*PFB*_ = 0, 0.1.
D. *u*_*a*_ = 0.01, *u*_*d*_ = 125, *u*_beta_ = 0.001, *d*_*a*_ = 20, *d*_*d*_ = 1, *k*_1_ = 0.01, *k*_2_ = 1, *k*_3_ = 1, *k*_4_ = 1, *k*_*b*1_ = 100, *k*_*b*2_ = 1, *k*_*b*3_ = 1, *k*_*b*4_ = 1, *k*_*ub*1_ = 1, *k*_*ub*2_ = 1, *k*_*ub*3_ = 1, *k*_*ub*4_ = 1, *A*_Total_ = 40, *P*_Total_ = 1, *K*_Total_ = 70, *X*_Total_ = 2, *k*_*PFB*_ = 0, 0.1.
E. *u*_*a*_ = 0.01, *u*_*d*_ = 10, *u*_beta_ = 0.001, *d*_*a*_ = 18, *d*_*d*_ = 12, *k*_1_ = 3, *k*_2_ = 60, *k*_3_ = 40, *k*_4_ = 12, *k*_*b*1_ = 40, *k*_*b*2_ = 2250, *k*_*b*3_ = 250, *k*_*b*4_ = 300, *k*_*ub*1_ = 1, *k*_*ub*2_ = 1, *k*_*ub*3_ = 1, *k*_*ub*4_ = 1, *A*_Total_ = 70, *P*_Total_ = 1, *K*_Total_ = 2, *X*_Total_ = 5, *k*_*PFB*_ = 6.1.
F. *u*_*a*_ = 0.01, *u*_*d*_ = 1, *u*_beta_ = 0.001, *d*_*a*_ = 200, *d*_*d*_ = 1, *k*_1_ = 1, *k*_2_ = 10, *k*_3_ = 1, *k*_4_ = 1, *k*_*b*1_ = 40, *k*_*b*2_ = 5, *k*_*b*3_ = 1, *k*_*b*4_ = 1, *k*_*ub*1_ = 1, *k*_*ub*2_ = 1, *k*_*ub*3_ = 1, *k*_*ub*4_ = 1, *A*_Total_ = 40, *P*_Total_ = 1, *K*_Total_ = 2, *X*_Total_ = 2, *k*_*PFB*_ = 0, 0.001, 0.002.

*Figure 3: Role of negative feedback from the fully modified substrate (*A*_*pp*_) in influencing intrinsic double-site modification network behaviour.*

A. *u*_*a*_ = 1, *u*_*d*_ = 20, *u*_beta_ = 0.01, *d*_*a*_ = 200, *d*_*d*_ = 1, *k*_1_ = 0.5, *k*_2_ = 1, *k*_3_ = 1, *k*_4_ = 1, *k*_*b*1_ = 30, *k*_*b*2_ = 10, *k*_*b*3_ = 1, *k*_*b*4_ = 1, *k*_*ub*1_ = 1, *k*_*ub*2_ = 1, *k*_*ub*3_ = 1, *k*_*ub*4_ = 1, *A*_Total_ = 40, *P*_Total_ = 1, *K*_Total_ = 3, *X*_Total_ = 5, *k*_*NFB*_ = 0, 10, 25, 50, 100.
B. *u*_*a*_ = 1, *u*_*d*_ = 10, *u*_beta_ = 0.001, *d*_*a*_ = 1, *d*_*d*_ = 1, *k*_1_ = 0.5, *k*_2_ = 0.1, *k*_3_ = 10, *k*_4_ = 1, *k*_*b*1_ = 100, *k*_*b*2_ = 100, *k*_*b*3_ = 1, *k*_*b*4_ = 1, *k*_*ub*1_ = 1, *k*_*ub*2_ = 1, *k*_*ub*3_ = 1, *k*_*ub*4_ = 1, *A*_Total_ = 40, *P*_Total_ = 1, *K*_Total_ = 40, *X*_Total_ = 2, *k*_*NFB*_ = 0, 10, 25, 50, 100.
C. *u*_*a*_ = 1, *u*_*d*_ = 10, *u*_beta_ = 0.001, *d*_*a*_ = 1, *d*_*d*_ = 1, *k*_1_ = 0.5, *k*_2_ = 1, *k*_3_ = 0.9, *k*_4_ = 50, *k*_*b*1_ = 1, *k*_*b*2_ = 1, *k*_*b*3_ = 200, *k*_*b*4_ = 3, *k*_*ub*1_ = 1, *k*_*ub*2_ = 1, *k*_*ub*3_ = 1, *k*_*ub*4_ = 1, *A*_Total_ = 40, *P*_Total_ = 1, *K*_Total_ = 10, *X*_Total_ = 4, *k*_*NFB*_ = 0, 5, 10, 15.
D. *u*_*a*_ = 1, *u*_*d*_ = 4, *u*_beta_ = 0.01, *d*_*a*_ = 2, *d*_*d*_ = 1, *k*_1_ = 1, *k*_2_ = 1, *k*_3_ = 0.9, *k*_4_ = 100, *k*_*b*1_ = 1, *k*_*b*2_ = 1, *k*_*b*3_ = 200, *k*_*b*4_ = 3, *k*_*ub*1_ = 1, *k*_*ub*2_ = 1, *k*_*ub*3_ = 1, *k*_*ub*4_ = 1, *A*_Total_ = 40, *P*_Total_ = 1, *K*_Total_ = 20, *X*_Total_ = 10, *k*_*NFB*_ = 0, 2, 4.
E. *u*_*a*_ = 0.01, *u*_*d*_ = 2, *u*_beta_ = 0.001, *d*_*a*_ = 200, *d*_*d*_ = 1, *k*_1_ = 1, *k*_2_ = 10, *k*_3_ = 1, *k*_4_ = 1, *k*_*b*1_ = 40, *k*_*b*2_ = 5, *k*_*b*3_ = 1, *k*_*b*4_ = 1, *k*_*ub*1_ = 0, *k*_*ub*2_ = 1, *k*_*ub*3_ = 1, *k*_*ub*4_ = 1, *A*_Total_ = 40, *P*_Total_ = 1, *K*_Total_ = 2, *X*_Total_ = 2, *k*_*NFB*_ = 0, 0.1, 0.25.

*Figure 4: Role of feedback from the partially modified substrate *A*_*p*_ in influencing intrinsic double-site modification network behaviour.*

A,E. *u*_*a*_ = 0.1, *u*_*d*_ = 100, *u*_beta_ = 0.01, *d*_*a*_ = 0.01, *d*_*d*_ = 1, *k*_1_ = 1, *k*_2_ = 1, *k*_3_ = 1, *k*_4_ = 1, *k*_*b*1_ = 1, *k*_*b*2_ = 1, *k*_*b*3_ = 1, *k*_*b*4_ = 1, *k*_*ub*1_ = 1, *k*_*ub*2_ = 1, *k*_*ub*3_ = 1, *k*_*ub*4_ = 1, *A*_Total_ = 40, *P*_Total_ = 1, *K*_Total_ = 5, *X*_Total_ = 1, *k*_*Ap*−*Act*_ = 0, 1, 2, 3, 5. *k*_*Ap*−*InAct*_ = 0, 50, 100, 250, 500.
B,F. *u*_*a*_ = 1, *u*_*d*_ = 10, *u*_beta_ = 0.001, *d*_*a*_ = 1, *d*_*d*_ = 1, *k*_1_ = 0.5, *k*_2_ = 0.1, *k*_3_ = 10, *k*_4_ = 1, *k*_*b*1_ = 100, *k*_*b*2_ = 100, *k*_*b*3_ = 1, *k*_*b*4_ = 1, *k*_*ub*1_ = 1, *k*_*ub*2_ = 1, *k*_*ub*3_ = 1, *k*_*ub*4_ = 1, *A*_Total_ = 40, *P*_Total_ = 1, *K*_Total_ = 40, *X*_Total_ = 2, *k*_*Ap*−*Act*_ = 0, 0.01, 0.02, 0.05. *k*_*Ap*−*InAct*_ = 0, 1, 5, 25.
C,G. *u*_*a*_ = 1, *u*_*d*_ = 10, *u*_beta_ = 0.001, *d*_*a*_ = 1, *d*_*d*_ = 1, *k*_1_ = 0.5, *k*_2_ = 1, *k*_3_ = 0.9, *k*_4_ = 50, *k*_*b*1_ = 1, *k*_*b*2_ = 1, *k*_*b*3_ = 200, *k*_*b*4_ = 3, *k*_*ub*1_ = 1, *k*_*ub*2_ = 1, *k*_*ub*3_ = 1, *k*_*ub*4_ = 1, *A*_Total_ = 40, *P*_Total_ = 1, *K*_Total_ = 10, *X*_Total_ = 4, *k*_*Ap*−*Act*_ = 0, 0.01, 0.05, 0.1. *k*_*Ap*−*InAct*_ = 0, 1, 2, 5.
D,H. *u*_*a*_ = 0.01, *u*_*d*_ = 2, *u*_beta_ = 0.001, *d*_*a*_ = 200, *d*_*d*_ = 1, *k*_1_ = 1, *k*_2_ = 10, *k*_3_ = 1, *k*_4_ = 1, *k*_*b*1_ = 40, *k*_*b*2_ = 5, *k*_*b*3_ = 1, *k*_*b*4_ = 1, *k*_*ub*1_ = 0, *k*_*ub*2_ = 1, *k*_*ub*3_ = 1, *k*_*ub*4_ = 1, *A*_Total_ = 40, *P*_Total_ = 1, *K*_Total_ = 2, *X*_Total_ = 2, *k*_*Ap*−*Act*_ = 0, 0.001, 0.005. *k*_*Ap*−*InAct*_ = 0, 0, 1, 0.25.

*Figure 5: Role of incoherent feed-forward from the fully modified substrate (*A*_*pp*_) in influencing intrinsic double-site modification network behaviour.*

A. *u*_*a*_ = 1, *u*_*d*_ = 1, *u*_beta_ = 0.01, *d*_*d*_ = 1, *d*_beta_ = 0.01, *k*_1_ = 1, *k*_2_ = 1, *k*_3_ = 1, *k*_4_ = 1, *k*_*b*1_ = 1, *k*_*b*2_ = 1, *k*_*b*3_ = 1, *k*_*b*4_ = 1, *k*_*ub*1_ = 1, *k*_*ub*2_ = 1, *k*_*ub*3_ = 1, *k*_*ub*4_ = 1, *A*_Total_ = 40, *P*_Total_ = 1, *K*_Total_ = 1, *k*_*iFFW*_ = 0, 0.1, 0.25, 0.5, 1, 2, 5, 10.
B. *u*_*a*_ = 1, *u*_*d*_ = 4, *u*_beta_ = 0.01, *d*_*d*_ = 2, *d*_beta_ = 0.01, *k*_1_ = 1, *k*_2_ = 1, *k*_3_ = 0.4, *k*_4_ = 1, *k*_*b*1_ = 1, *k*_*b*2_ = 1, *k*_*b*3_ = 1, *k*_*b*4_ = 1, *k*_*ub*1_ = 1, *k*_*ub*2_ = 1, *k*_*ub*3_ = 1, *k*_*ub*4_ = 1, *A*_Total_ = 40, *P*_Total_ = 1.5, *K*_Total_ = 1, *k*_*iFFW*_ = 0, 0.1, 0.25, 0.5, 1, 2.
C. *u*_*a*_ = 1, *u*_*d*_ = 8, *u*_beta_ = 0.01, *d*_*d*_ = 4, *d*_beta_ = 0.01, *k*_1_ = 0.1, *k*_2_ = 1, *k*_3_ = 1, *k*_4_ = 1, *k*_*b*1_ = 40, *k*_*b*2_ = 10, *k*_*b*3_ = 1, *k*_*b*4_ = 1, *k*_*ub*1_ = 1, *k*_*ub*2_ = 1, *k*_*ub*3_ = 1, *k*_*ub*4_ = 1, *A*_Total_ = 40, *P*_Total_ = 1.1, *K*_Total_ = 1, *k*_*iFFW*_ = 0, 0, 05, 0.1, 0.25, 1.
D. *u*_*a*_ = 1, *u*_*d*_ = 5, *u*_beta_ = 0.01, *d*_*d*_ = 2, *d*_beta_ = 0.01, *k*_1_ = 0.8, *k*_2_ = 1, *k*_3_ = 1, *k*_4_ = 40, *k*_*b*1_ = 10, *k*_*b*2_ = 1, *k*_*b*3_ = 100, *k*_*b*4_ = 1, *k*_*ub*1_ = 1, *k*_*ub*2_ = 1, *k*_*ub*3_ = 1, *k*_*ub*4_ = 1, *A*_Total_ = 40, *P*_Total_ = 20, *K*_Total_ = 15, *k*_*iFFW*_ = 0, 0.01, 0.05.
E. *u*_*a*_ = 1, *u*_*d*_ = 1, *u*_beta_ = 0.01, *d*_*d*_ = 1, *d*_beta_ = 0.01, *k*_1_ = 0.8, *k*_2_ = 2, *k*_3_ = 1, *k*_4_ = 10, *k*_*b*1_ = 3, *k*_*b*2_ = 1, *k*_*b*3_ = 4, *k*_*b*4_ = 1, *k*_*ub*1_ = 1, *k*_*ub*2_ = 1, *k*_*ub*3_ = 1, *k*_*ub*4_ = 1, *A*_Total_ = 40, *P*_Total_ = 10, *K*_Total_ = 8, *k*_*iFFW*_ = 0, 0.1, 0.15.
F. *u*_*a*_ = 1, *u*_*d*_ = 3, *u*_beta_ = 0.01, *d*_*d*_ = 75, *d*_beta_ = 1, *k*_1_ = 0.01, *k*_2_ = 1, *k*_3_ = 0.01, *k*_4_ = 1, *k*_*b*1_ = 1, *k*_*b*2_ = 10, *k*_*b*3_ = 25, *k*_*b*4_ = 1, *k*_*ub*1_ = 1, *k*_*ub*2_ = 1, *k*_*ub*3_ = 1, *k*_*ub*4_ = 1, *A*_Total_ = 40, *P*_Total_ = 5, *K*_Total_ = 2, *k*_*iFFW*_ = 0, 5, 25, 40.
G. *u*_*a*_ = 3, *u*_*d*_ = 4, *u*_beta_ = 0.01, *d*_*d*_ = 100, *d*_beta_ = 1, *k*_1_ = 0.01, *k*_2_ = 1, *k*_3_ = 0.05, *k*_4_ = 1, *k*_*b*1_ = 1, *k*_*b*2_ = 1, *k*_*b*3_ = 1, *k*_*b*4_ = 1, *k*_*ub*1_ = 1, *k*_*ub*2_ = 1, *k*_*ub*3_ = 1, *k*_*ub*4_ = 1, *A*_Total_ = 40, *P*_Total_ = 5, *K*_Total_ = 2, *k*_*iFFW*_ = 0, 0.25.

*Figure 6: Role of coherent feed-forward from the fully modified substrate (*A*_*pp*_) in influencing intrinsic double-site modification network behaviour.*

A. *u*_*a*_ = 1, *u*_*d*_ = 1, *u*_beta_ = 0.01, *d*_*d*_ = 1, *d*_beta_ = 0.01, *k*_1_ = 1, *k*_2_ = 0.1, *k*_3_ = 1.5, *k*_4_ = 1, *k*_*b*1_ = 1, *k*_*b*2_ = 1, *k*_*b*3_ = 1, *k*_*b*4_ = 1, *k*_*ub*1_ = 1, *k*_*ub*2_ = 1, *k*_*ub*3_ = 1, *k*_*ub*4_ = 1, *A*_Total_ = 40, *P*_Total_ = 1, *K*_Total_ = 1, *k*_*cFFW*_ = 0, 0.1, 0.25, 1, 2.
B. *u*_*a*_ = 1, *u*_*d*_ = 10, *u*_beta_ = 0.01, *d*_*d*_ = 1, *d*_beta_ = 1, *k*_1_ = 1, *k*_2_ = 0.1, *k*_3_ = 100, *k*_4_ = 1, *k*_*b*1_ = 1, *k*_*b*2_ = 1, *k*_*b*3_ = 1, *k*_*b*4_ = 1, *k*_*ub*1_ = 1, *k*_*ub*2_ = 1, *k*_*ub*3_ = 1, *k*_*ub*4_ = 1, *A*_Total_ = 40, *P*_Total_ = 1, *K*_Total_ = 70, *k*_*cFFW*_ = 0, 0.1, 0.25, 0.5, 1.
C. *u*_*a*_ = 1, *u*_*d*_ = 3, *u*_beta_ = 0.01, *d*_*d*_ = 0.75, *d*_beta_ = 0.01, *k*_1_ = 0.01, *k*_2_ = 1, *k*_3_ = 0.01, *k*_4_ = 1, *k*_*b*1_ = 1, *k*_*b*2_ = 10, *k*_*b*3_ = 25, *k*_*b*4_ = 1, *k*_*ub*1_ = 1, *k*_*ub*2_ = 1, *k*_*ub*3_ = 1, *k*_*ub*4_ = 1, *A*_Total_ = 40, *P*_Total_ = 5, *K*_Total_ = 2, *k*_*cFFW*_ = 0, 5, 25.
D. *u*_*a*_ = 1, *u*_*d*_ = 5, *u*_beta_ = 0.01, *d*_*d*_ = 2, *d*_beta_ = 0.01, *k*_1_ = 0.8, *k*_2_ = 1, *k*_3_ = 1, *k*_4_ = 40, *k*_*b*1_ = 10, *k*_*b*2_ = 1, *k*_*b*3_ = 100, *k*_*b*4_ = 1, *k*_*ub*1_ = 1, *k*_*ub*2_ = 1, *k*_*ub*3_ = 1, *k*_*ub*4_ = 1, *A*_Total_ = 40, *P*_Total_ = 20, *K*_Total_ = 15, *k*_*cFFW*_ = 0, 50, 250.
E. *u*_*a*_ = 1, *u*_*d*_ = 1, *u*_beta_ = 0.01, *d*_*d*_ = 1, *d*_beta_ = 0.01, *k*_1_ = 0.1, *k*_2_ = 0.5, *k*_3_ = 1, *k*_4_ = 20, *k*_*b*1_ = 5, *k*_*b*2_ = 1, *k*_*b*3_ = 25, *k*_*b*4_ = 1, *k*_*ub*1_ = 1, *k*_*ub*2_ = 1, *k*_*ub*3_ = 1, *k*_*ub*4_ = 1, *A*_Total_ = 40, *P*_Total_ = 15, *K*_Total_ = 15, *k*_*cFFW*_ = 0, 1.
F. *u*_*a*_ = 1, *u*_*d*_ = 5, *u*_beta_ = 0.01, *d*_*d*_ = 10, *d*_beta_ = 1, *k*_1_ = 0.01, *k*_2_ = 1, *k*_3_ = 0.05, *k*_4_ = 1, *k*_*b*1_ = 2, *k*_*b*2_ = 1, *k*_*b*3_ = 1, *k*_*b*4_ = 1, *k*_*ub*1_ = 1, *k*_*ub*2_ = 1, *k*_*ub*3_ = 1, *k*_*ub*4_ = 1, *A*_Total_ = 40, *P*_Total_ = 5, *K*_Total_ = 2, *k*_*cFFW*_ = 0, 2.
G. *u*_*a*_ = 1, *u*_*d*_ = 5, *u*_beta_ = 0.01, *d*_*d*_ = 0.1, *d*_beta_ = 0.01, *k*_1_ = 0.1, *k*_2_ = 1, *k*_3_ = 0.9, *k*_4_ = 100, *k*_*b*1_ = 2, *k*_*b*2_ = 1, *k*_*b*3_ = 100, *k*_*b*4_ = 1, *k*_*ub*1_ = 1, *k*_*ub*2_ = 1, *k*_*ub*3_ = 1, *k*_*ub*4_ = 1, *A*_Total_ = 40, *P*_Total_ = 3, *K*_Total_ = 7, *k*_*cFFW*_ = 0, 5.
